# Decoding MAPT Exon 10 Mis-Splicing in FTDP-17: From Pathogenic Mechanisms and Experimental Models to Molecular Therapies

**DOI:** 10.3390/genes17091150

**Published:** 2026-09-19

**Authors:** Giuseppina Covello

**Affiliations:** Department of Biology, University of Padua, 35131 Padua, Italy; giuseppina.covello@unipd.it; Tel.: +39-049-8277464

**Keywords:** MAPT exon 10, alternative splicing, tau 3R/4R ratio, FTDP-17, antisense oligonucleotides (ASOs), RNA interference (siRNA), small-molecule splicing modulators, human iPSC models, cerebral organoids, intrathecal drug delivery

## Abstract

Frontotemporal Dementia and Parkinsonism linked to chromosome 17 (FTDP-17) is a rare, early-onset, autosomal-dominant neurodegenerative tauopathy caused by mutations in the *Microtubule-Associated Protein Tau* (*MAPT*) gene. A subset of these mutations selectively disrupts the normal alternative splicing of MAPT exon 10, altering the physiological ratio of 3-Repeat (3R) and 4-Repeat (4R) tau isoforms; this imbalance, driving pathological tau aggregation and progressive neurodegeneration, is a pathogenic hallmark of FTDP-17. This review explores the molecular mechanisms regulating exon 10 splicing, focusing on how exonic and intronic splicing mutations destabilise mRNA structures or alter splicing factor binding, and discusses how these changes can cause disease symptoms in experimental models. It describes different research methods for studying splicing issues, including minigene reporters, cell lines, human induced pluripotent stem cell (iPSC)-derived neurons, brain organoids and mouse models that naturally express only 4R tau. It also covers potential therapeutic approaches applicable to restoring the normal 3R/4R tau ratio. These include Antisense Oligonucleotides (ASOs), RNA interference (siRNA), Small-Molecule Splicing modulators (SMCs), Spliceosome-Mediated RNA *trans*-splicing (SMaRT), CRISPR-based transcript engineering, genome editing and RNA stem-loop binders. Additionally, the review highlights recent Phase 2 trial results, including the Diranersen (BIIB080) tau-lowering ASO, the development of MAPT-targeted RNA therapies, new tau-PET and plasma p-tau biomarkers, and innovative strategies to deliver CNS treatments, including non-invasive approaches. By integrating advances in RNA biology, disease modelling and targeted therapies, the review outlines potential future strategies for treating FTDP-17 and other tau-related disorders.

## 1. Introduction

Frontotemporal Lobar Degeneration (FTLD) is a heterogeneous group of neurodegenerative disorders characterised by progressive atrophy of the frontal and temporal lobes and a variety of clinical and neuropathological features. As the second most common cause of early-onset dementia in individuals under 65, FTLD typically causes profound changes in personality, social behaviour, and cognitive function, alongside progressive language deficits such as primary progressive aphasia. FTLD syndromes are classified by the predominant protein found within intracellular inclusions. The Microtubule-Associated Protein Tau, encoded by the *MAPT* gene located on chromosome 17q21 (OMIM #157140) [1], is a primary cause of the disease. It has been the focus of research for over a century, and a comprehensive historical overview of tau pathology was published [2].

A major advance in our understanding of the molecular pathogenesis of Frontotemporal Lobar Degeneration (FTLD) occurred in 1998. Genetic linkage studies mapped familial cases of Frontotemporal Lobar Dementia (FTLD) linked to Parkinsonism to chromosome 17q21–22.

These studies led to the identification of pathogenic mutations in the *Microtubule-Associated Protein Tau* (*MAPT*) gene that, alone, trigger toxic tau filament aggregation, hyperphosphorylation, and neuronal death, as well as the clinical–pathological entity Frontotemporal Dementia and Parkinsonism linked to chromosome 17 (FTDP-17) (OMIM #600274) [3,4]. This identification provided important evidence that tau dysfunction can causally contribute to neurodegeneration. In contrast, the precise contribution of intracellular tau aggregates, such as neurofibrillary tangles (NFTs), to neuronal degeneration remains under investigation.

Therefore, *MAPT* is a central disease gene across a broad spectrum of tauopathies, and FTDP-17 is a key study model for this group.

Notably, an imbalanced 3R/4R tau isoform ratio is not unique to genetic FTDP-17 disorder: sporadic tauopathies such as Progressive Supranuclear Palsy (PSP) and Corticobasal Degeneration (CBD) are characterised by predominantly 4R-tau pathology. In contrast, Pick’s disease is defined by 3R-tau inclusions. Additionally, each disorder shows distinct cell-type-specific patterns of tau aggregation in neurons versus glia [5,6]. This imbalance in tau isoforms in sporadic diseases extends beyond the 3R/4R (exon 10) axis. A coordinated splicing programme linking exons 2 and 10 is differentially disrupted in PSP and Alzheimer’s disease. In PSP, dysregulated expression of exon 2 splicing regulators drives the accumulation of 1N4R tau isoforms, whereas in AD, it drives the accumulation of 0N isoforms. This different regulatory system indicates that distinct tauopathies can arise from the dysregulation of the same broader MAPT splicing network in a disease-specific manner, rather than from a single, uniform mechanism [7]. These findings suggest that changes in the pre-mRNA MAPT splicing network result in distinct tau isoform profiles across tauopathies.

A recent overview of tau’s diverse cellular functions in the central nervous system (CNS) was provided, including axonal microtubule stability and dynamics, intracellular transport regulation and synaptic signalling, stress granule biology, and the mechanisms driving its pathological aggregation in tauopathies [1,8,9]. The *MAPT* gene spans approximately 100 kb on the long arm of chromosome 17 at 17q21.31 [8] and comprises 16 exons. However, the primary transcript comprises only 13 exons since exons 4A, 6 and 8 are not transcribed in humans, while exons -1 and 14 are transcribed but not translated [10]. Exons 1, 4, 5, 7, 9, 11, 12 and 13 are constitutive, whereas exons 2, 3 and 10 are alternatively spliced in the adult human brain [10,11]. Interestingly, exon 3 never arises independently of exon 2, a correlation whose molecular basis remains incompletely understood [10]. Alternative splicing of these three exons generates six distinct CNS tau isoforms ranging from 352 to 441 amino acids [1]. Exons 2 and 3 encode N-terminal inserts, resulting in 0N, 1N, and 2N tau variants. These are expressed in markedly different proportions in the adult human brain (approximately 37%, 54%, and 9% of total tau, respectively) and appear to serve different physiological functions during development [12,13].

Importantly, alternative splicing of exon 10, a 93-base-pair sequence located between exons 9–11, determines the number of microtubule-binding repeats (MTBRs) in tau, resulting in isoforms containing either three (3R) or four (4R) imperfect C-terminal microtubule-binding repeat domains (MTBRs) [14]. The additional 21-amino-acid repeat encoded by exon 10 gives 4R tau isoforms with higher microtubule-binding affinity and distinct assembly kinetics compared to 3R tau [15]. In the healthy adult brain, the splicing machinery maintains a near-equimolar (~1:1) ratio between 3R and 4R tau isoforms across most brain regions. This ratio is essential for normal microtubule dynamics, vesicle transport and axonal integrity [14,15]. By contrast, the human foetal brain exclusively expresses the shortest 0N3R isoform, reflecting a precise developmental change from 3R tau to the balanced adult profile [16]. Researchers recently described a non-aggregative tau splicing isoform, which they found to be decreased in Alzheimer’s disease. This research provides further evidence that the functional consequences of MAPT alternative splicing extend beyond the canonical 3R/4R axis [17].

Interestingly, the regulation of exon 10 splicing is not conserved between species, despite the high sequence conservation of the exon itself. The mature adult mouse brain predominantly expresses tau as the 4R isoform at the whole-tissue level, because all murine tau transcripts constitutively include exon 10. In contrast, adult human brains maintain the balanced 3R/4R profile described above [16,18]. This cross-species divergence in splicing outcome, driven by structural differences in the regulatory sequences flanking exon 10 rather than in the exon itself [18], underlies the central modelling challenge discussed throughout this review and motivates the use of humanised rather than wild-type rodent models. Any genetic or epigenetic perturbation that shifts the human 3R/4R ratio towards either isoform is sufficient to cause pathological tau aggregation, as observed in FTDP-17 and related tauopathies.

Moreover, a complex network of *cis*-acting regulatory elements and *trans*-acting splicing factors regulates the alternative splicing of MAPT exon 10 pre-mRNA. Exon 10 has weak splice sites, and its recognition by the spliceosome is severely limited by a highly conserved secondary stem-loop structure at the junction between the 3′ end of exon 10 and the 5′ end of intron 10 [19]. Under physiological conditions, this hairpin structure sequesters the 5′ splice site, restricting U1 small nuclear ribonucleoprotein (snRNP) binding and inducing exon skipping (production of 3R tau) [19,20].

The stability of this stem-loop is further modulated by *trans*-acting factors that bind to exonic splicing enhancers (ESEs) or silencers (ESSs). SR (serine/arginine-rich) proteins, such as TRA2β, SRSF1 and SRSF2, bind to ESE sequences within exon 10, thereby promoting its inclusion [21]. In contrast, SRSF4 and heterogeneous nuclear ribonucleoproteins (hnRNPs), including hnRNP G and hnRNP A1, act as repressors, favouring exon 10 exclusion. The effects of these splicing factors are themselves subject to upstream regulation. Indeed, CLK2 phosphorylates SR proteins, including TRA2β, and the CLK2/TRA2β signalling has been reported in sporadic Alzheimer’s disease. Therefore, the kinase regulation of the splicing-factor network controls the 3R/4R ratio via indirect mechanisms beyond the genetically driven mechanisms characteristic of FTDP-17 [22]. The question of whether alternative splicing dysregulation is a cause or consequence of neurodegeneration in sporadic Alzheimer’s disease is still being debated [23].

Comparative genomic analyses across primate species have identified muscleblind-like splicing regulator 2 (MBNL2) as an additional regulator of MAPT exon 10 splicing [24]. MBNL2, a *trans*-acting protein, binds distal intronic sites, distinct from the intron 10 stem-loop and the ESE/ESS elements described above, and may modulate exon 10 inclusion through these regulatory sites [24]. According to the data, MBNL2-binding sites could represent an additional targetable regulatory region, distinct from the intron 10 stem-loop and the canonical ESE/ESS network. In this context, the Cas13d (dCas13d) guide RNA complex system can be used to reduce exon 10 inclusion *in vitro*, providing proof of concept for CRISPR-dCas13d-mediated transcript engineering.

Although we now better understand the genetic and molecular processes behind MAPT exon 10 mis-splicing, translating these insights into disease-relevant preclinical models remains challenging. A significant species-specific limitation is that adult rodents predominantly express 4R tau and fail to recapitulate the physiological human 3R/4R tau ratio or its pathological alteration observed in FTDP-17 [25]. Consequently, conventional rodent models have limited capacity to replicate human MAPT exon 10 splicing and to facilitate the development and validation of splicing-corrective therapies. Recent advances, including human *MAPT*-based mouse models, patient-derived iPSCs and iPSC-derived neurons, three-dimensional (3D) cerebral organoids, and human organotypic brain slices, have progressively expanded the experimental repertoire and provided complementary platforms for bridging the gap between mechanistic studies and human disease biology [26,27]. In addition, therapeutic strategies targeting exon 10, including Antisense Oligonucleotides (ASOs), Small-Molecule Splicing modulators (SMCs), RNA *trans*-splicing (SMaRT), and CRISPR-based transcript and genome editing, offer novel approaches to restore the physiological 3R/4R tau ratio. Against this background, this review summarises the emerging experimental models and therapeutic strategies targeting MAPT exon 10 splicing correction, focusing on their biological relevance, limitations, and potential as new therapeutic approaches for FTDP-17 and tauopathies.

## 2. *MAPT* Exon 10 Alternative Splicing Mutations in FTDP-17

However, the same splicing regulatory network can be altered in either direction, depending on which *cis*-element is precisely altered. Of the 23 FTDP-17 mutations reported in the literature and summarised in Table 1, the vast majority (17 out of 23) promote exon 10 inclusion and consequently increase 4R-tau levels, while a smaller subset (5 out of 23) induces exon 10 exclusion and increased production of 3R-tau isoforms; the remaining variant, E9+33, has a direction of effect that has not been fully characterised.

These pathogenic mutations disrupt this regulatory network in two ways, underlining the combinatorial nature of exon 10 splicing control [31]. Indeed, intronic mutations (e.g., IVS10+13, IVS10+16, IVS10+14, IVS10+11, and IVS10+3) destabilise the intron 10 stem-loop by unwinding the hairpin. The U1 snRNP unimpeded access to the 5′ splice site results in massive exon 10 hyper-inclusion [3,4,19,20]. The exonic mutations (e.g., N279K, S305N, and L284L) instead alter the local ESE/ESS landscape, with most creating or strengthening positive splicing enhancers that drive exon 10 inclusion [21,30]. Analysis of both missense and silent FTDP-17 exonic mutations has shown that they act by disrupting multiple distinct alternative splicing regulatory elements rather than a single dominant enhancer. In contrast, the exonic ΔK280 deletion operates through the opposite mechanism. It disrupts a purine-rich ESE and causes pathological exon 10 exclusion, resulting in an excess of 3R tau. Consequently, it is used in some hiPSC platforms as a control background for the isoform imbalance [33]. 

The N279K mutation [35,36] caused a significant alteration in exon 10, resulting in increased inclusion of the 4R tau isoform, accumulation of 4R tau, progressive conformational change, and self-assembly of tau into toxic oligomers and insoluble filaments (NF) (NFTs). The deposition of these filaments [37] causes Frontotemporal Lobar Degeneration (FTLD) [1,4].

Beyond their effects on splicing per se, specific FTDP-17 mutations have also been shown to impair the normal microtubule-binding and -assembly functions of the resulting mutant tau isoforms in different ways. Therefore, the individual mutations can combine a splicing-driven isoform imbalance with isoform-intrinsic functional deficits [34]. These observations indicate the complexity and bidirectionality of the regulatory mechanisms that control exon 10 splicing, emphasising the necessity for experimental models that accurately mimic the human 3R/4R splicing pattern.

## 3. Experimental Models for MAPT Exon 10 Splicing: From *In Vitro* to *In Vivo* Systems

**Figure 1:**
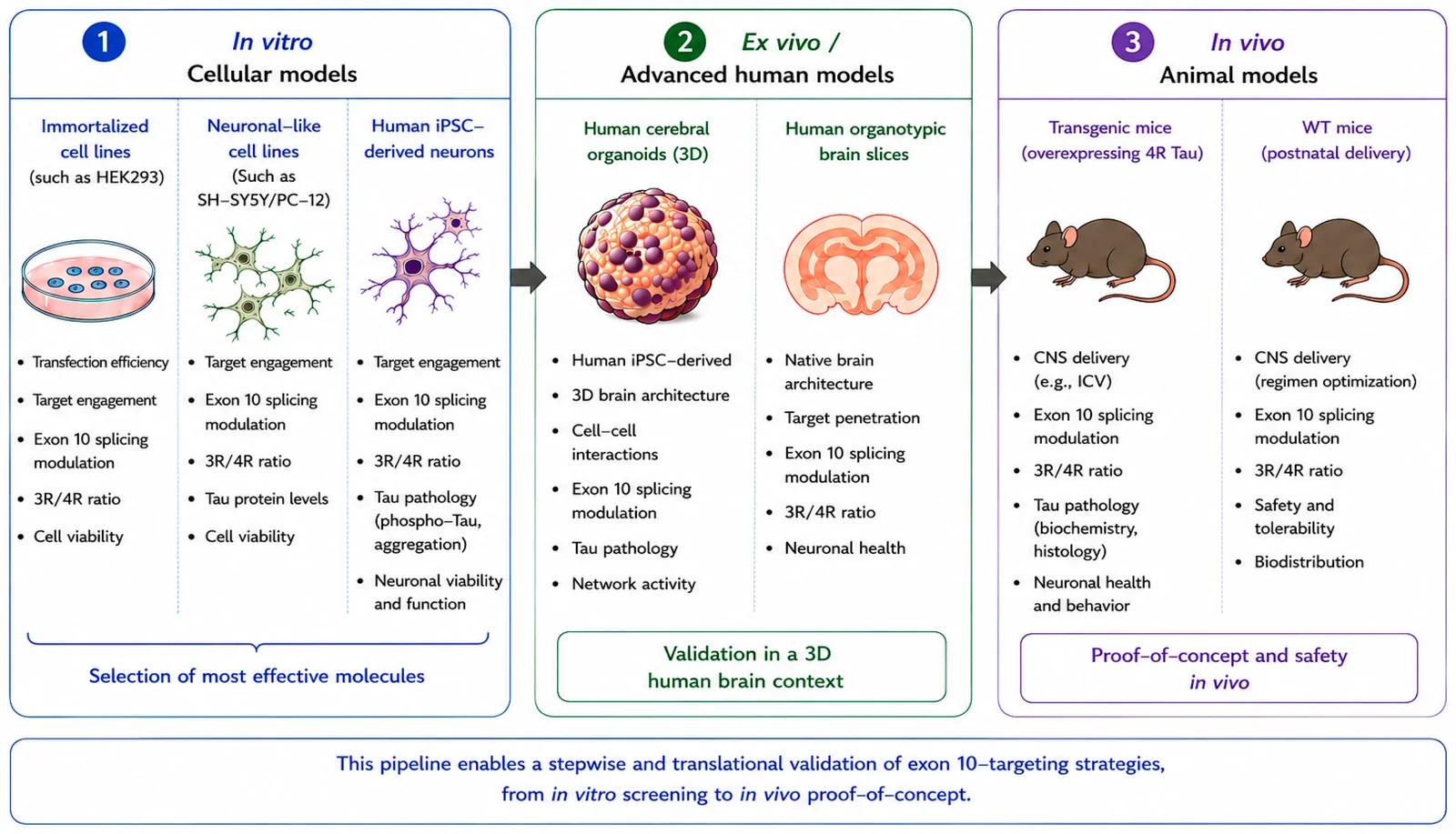
Overview of experimental platforms used to study MAPT exon 10 alternative splicing and test therapies that target exon 10. (1) Cellular models include immortalised cell lines like HEK293, neuronal-like lines such as SH-SY5Y and PC12, and patient-derived human iPSC-neurons that support disease-relevant genetic backgrounds. (2) Human iPSC-derived cerebral organoids and *ex vivo* organotypic brain slices offer more physiologically relevant human models for studying splicing regulation, tau pathology, and how therapies work. (3) *In vivo* mouse models include transgenic mice that overexpress 4R tau, which are used to test therapy effectiveness, tau pathology, and behaviour. Wild-type mice help optimise CNS delivery, safety, and biodistribution.

Investigating and correcting MAPT exon 10 splicing requires a carefully staged pipeline of experimental platforms, each offering a different balance of throughput, physiological relevance, and proximity to human disease. This section provides a comprehensive, comparative overview of this pipeline. It spans immortalised cell lines optimised for High-Throughput Screening, patient-derived human iPSC-neurons that maintain disease-related genetic backgrounds, and advanced three-dimensional human organoids and *ex vivo* organotypic brain slices that offer complex tissue environments. Finally, we review *in vivo* animal models, which are useful for evaluating splicing modulation, therapeutic efficacy, biodistribution and safety. Figure 1 summarises this overall pipeline.

### 3.1. Cell Models for Studying MAPT Exon 10 Splicing

Before stem cell technologies, biochemical systems and immortalised cell lines were widely used to investigate molecular mechanisms underlying disruption of the 3R/4R tau ratio, especially the regulation of MAPT exon 10 alternative splicing. Their rapid proliferation and ease of genetic manipulation also offer complementary advantages for biological studies and initial stages of drug discovery [38,39]. 

Common cellular models used include immortalised cell lines for *MAPT* exon 10 minigene assays. The transient minigene assay is widely used *in vivo* to analyse alternative splicing defects, including intrinsic *cis*-acting elements and *trans*-acting factors, and to confirm whether a candidate genetic variant causes exon skipping before classification as pathogenic [40,41,42]. A *MAPT* minigene includes exon 10 with its flanking intronic sequences (intron 9 and intron 10), and the typical cell lines used as a backbone to transfect this minigene are HEK293/HEK293T or HeLa cells (Figure 1, Panel 1) [36,38]. Human embryonic kidney (HEK293) cells are particularly well-suited to these reporter assays because they are easy to transfect at high efficiency, proliferate rapidly and tolerate the stable integration of large minigene constructs. They also do not express high levels of endogenous MAPT mRNA, providing a biologically neutral background without confounding contributions from an endogenous transcript [38].

Indeed, studies carried out on these cell lines revealed a link between exon 10 splicing defects and intronic mutations, such as IVS10+16 [36]. Using a set of intron-deletion minigene constructs spanning exons 9–11 and an early luciferase-based reporter system, it was further confirmed that correct exon 10 splicing requires a minimum intronic distance between exons 10 and 11 and that the SR protein SRp20 acts as a dosage-dependent repressor capable of promoting exon 10 skipping, even in constructs carrying FTDP-17-associated mutations [43]. Furthermore, HEK293T cell lines that are stably transfected with an integrated *MAPT* minigene have become the standard first-tier platform for compound screening in SMC drug discovery pipelines [44]. Despite their inherently simple design and lack of neuronal-specific splicing context, modern dual-colour fluorescent frame-shift reporters [45] have enabled minigene assays to serve as a robust platform for High-Throughput Screening (HTS) of chemical libraries, as exemplified by the HCS-Splice assay. This minigene uses a two-colour GFP/RFP *MAPT* exon 10 reporter that has been validated in SH-SY5Y cells [46].

To bridge the gap between heterologous cell lines and human neural biology, researchers often use the SH-SY5Y human neuroblastoma cell line (Figure 1, Panel 1) [47]. This cell line, under endogenous conditions, expresses all six human tau isoforms [39] and predominantly expresses 3R tau in an undifferentiated state, thereby mimicking fetal brain development. Upon differentiation, the cells upregulate 4R tau, providing a dynamic model of the physiological shift towards the adult 1:1 ratio [47,48]. Therefore, improvements to SH-SY5Y cell differentiation protocols in multi-well formats, combined with high-throughput screening technologies, make this cell model useful for neurite outgrowth assays and, especially, for screening applications beyond splicing reporters [49]. Moreover, SH-SY5Y cells, stably or transiently transfected with mutant *MAPT* minigene constructs (e.g., P301L or N279K), allowed investigators to examine the impact of a shifted 3R/4R isoform ratio on cytotoxicity, tau hyperphosphorylation, and caspase-3-mediated apoptosis. Additionally, expressing each of the six tau isoforms individually in these cells has revealed isoform-specific effects at the cellular level [50].

A useful model for axonal transport and cytoskeletal dynamics is the rat pheochromocytoma cell line (PC-12) [51]. They can retain the ability to undergo alternative splicing, producing both 3R and 4R tau proteins depending on their state of differentiation. Historically, they have played a key role in validating early Antisense Oligonucleotide molecules designed to mask the 5′ and 3′ splice junctions of exon 10 [52]. When human MAPT exon 10 isoforms are overexpressed in PC12 cells, researchers can study how an imbalanced 3R/4R ratio affects NGF-induced neurite outgrowth, disrupts kinesin-driven anterograde transport, and makes the cells more vulnerable to oxidative stress [53]. Although SH-SY5Y and PC12 cells are highly stable and easy to work with, their cancerous origins and abnormal chromosome numbers limit their ability to mimic the long-term, age-related neurodegeneration seen in human patients.

In addition to the standard human and rat lines, the mouse motor neuron-like hybrid line NSC-34 is a validated model of motoneuronal cholinergic identity that can be differentiated [54]. It has been crucial in mapping the broader pathological network of upstream splicing regulators shared by tauopathies and Amyotrophic Lateral Sclerosis (ALS)/Frontotemporal Lobar Degeneration (FTLD) [55]. Mechanistic studies in NSC-34 cells have shown that the RNA-binding proteins Fused in Sarcoma (FUS) and splicing factor Proline- and Glutamine-Rich (SFPQ) bind cooperatively to new MAPT transcripts. Disrupting the FUS/SFPQ complex results in immediate over-inclusion of exon 10. This regulatory mechanism proves the role of these non-tau nuclear proteins in driving 4R-tau toxicity [55].

A useful intermediate 2D cellular platform between fully immortalised non-neuronal lines and patient-derived hiPSC neurons is the ReNcell VM human neural progenitor cells. The ReNcell VM is able to differentiate into a mixed population of electrophysiologically active neurons, astrocytes, and oligodendrocytes upon growth factor withdrawal [56]. Unlike HEK293 or SH-SY5Y cells, differentiated ReNcell VM neurons endogenously express tau protein at detectable levels. The number of tau-positive cells increases progressively during differentiation, making this line a tractable, non-patient-specific human model for studying endogenous tau expression, phosphorylation, and activity-dependent release in a genetically normal (non-mutant) background [57]. Researchers use ReNcell VM as a bridging tool to test compounds and RNA therapies before moving to patient-derived hiPSC neurons. It is chosen for its human origin, scalability, and endogenous MAPT expression levels, which HEK293 and SH-SY5Y lines only partly provide.

### 3.2. Human iPSC-Derived Neuronal Models

Although immortalised cell lines are helpful for high-throughput biochemical screening in FTDP-17 studies, they do not have the same genomic structure as human patients and do not fully represent the neurodegenerative process [55]. Human induced pluripotent stem cells (iPSCs) are generated by reprogramming adult cells with specific transcription factors and overcome a key limitation of rodent models (Figure 1, Panel 1) [58]. For example, the mature mouse brain predominantly expresses 4R tau, as murine MAPT transcripts constitutively include exon 10 [25,59,60]; a minor exception occurs in actively neurogenic niches, such as the hippocampal dentate gyrus and subventricular zone, where newly generated adult-born neurons transiently express 3R tau before switching to 4R as they mature [59,60]. However, 3R tau is mainly observed during embryonic and early postnatal development.

The strengths and weaknesses of using hiPSCs for reprogramming, neuronal differentiation, and disease-related phenotyping in FTDP-17 research have been comprehensively reviewed [61].

hiPSC lines derived from patients with autosomal dominant FTDP-17 mutations, such as IVS10+16, IVS10+14, IVS10+11, IVS10+3, N279K, S305N, S305I, S305S, or the ΔK280 deletion, or those engineered using CRISPR/Cas9, were turned into two-dimensional (2D) layers of cortical or motor neurons. This process was accomplished using standard protocols for forced expression of Neurogenin-2 (NGN2) or dual-SMAD inhibition [30,55]. Under physiological conditions, immature human neurons express only the shortest 3R isoform (0N3R), progressing slowly and progressively toward an adult human 1:1 ratio over several months *in vitro*.

In neurons carrying intronic mutations (IVS10+16, IVS10+14, IVS10+11 and IVS10+3), the pathology arises from destabilisation of the exon 10–intron 10 hairpin structure, increasing accessibility of the 5′ splice site to the U1 snRNP. In neurons with the IVS10+14 variant specifically, this splicing defect is linked to calcium dysregulation as an early contributor to neurodegeneration [62]. In addition, IVS10+16 neurons display pathophysiological hyperexcitability due to the accumulation of 4R tau [63].

Interestingly, unlike its more prevalent intronic counterparts, no hiPSC-based model of the rarer IVS10+13 variant has been reported in the peer-reviewed literature. Since this mutation was originally described in a single family and is associated with a distinct clinical manifestation characterised by apathy rather than disinhibition, generating a dedicated hiPSC line would clarify whether its cellular and splicing phenotypes diverge from those of IVS10+16 neurons.

hiPSC carrying exonic mutations (N279K, S305N, S305I and S305S) recapitulate a different mechanism. Here, the sequence alteration generates or reinforces aberrant ESEs that hyper-recruit positive *trans*-acting factors, such as Tra2β [28]. In N279K neural stem cells specifically, this splicing defect has also been associated with impaired subcellular vesicle trafficking and elevated cellular stress, independently of overt 4R tau aggregation [64]. Furthermore, studies using heterologous expression have shown that N279K tau is more likely to be found in the nucleus than in the cytosol. This different localisation suggests an extra gain-of-function effect beyond the splicing defect [65].

Notably, while most variants increase exon 10 inclusion, the exonic ΔK280 deletion acts through an opposite mechanism. It disrupts a critical purine-rich ESE, resulting in exon 10 exclusion and an increase in the 3R tau isoform level. Researchers therefore use it across several hiPSC platforms as a control background to balance opposing isoforms [30].

Across these hiPSC models, the pathological shift toward increased 4R tau can progressively emerge during prolonged differentiation (approximately 100–365 days), reflecting the gradual maturation of the exon 10 splicing program and, in mutant models, the additional *cis*-acting effect of FTDP-17 variants at the *MAPT* locus [30,55,66].

Regardless of which hiPSC neuron models are generated, the resulting altered 3R/4R ratio leads to the development of early neurodegenerative phenotypes, including impaired microtubule stabilisation, defective mitochondrial and axonal transport, altered intracellular dynamics, increased oxidative stress and localised synaptic loss [30,55].

Further independent hiPSC-neuron studies across multiple *MAPT* mutations have reported tau fragmentation, increased phospho-tau immunoreactivity, decreased neurite outgrowth and activation of the unfolded protein response. These studies have also identified disease-associated transcriptomic signatures relative to isogenic controls [67]. These patient-derived monolayer models are a useful way to study early biological changes.

They also provide a gold-standard test for checking how splicing-corrective therapies work in different patient-specific genetic backgrounds [67]. Nevertheless, although 2D hiPSC-neurons retain an overall foetal-like splicing profile, some maturation-associated increase in 4R tau can occur during differentiation over time, providing a useful readout for studying disease mechanisms, mutation effects, and the pharmacological activity of splicing-targeting approaches such as siRNAs or ASOs [55,64,66]. However, this readout emerges only after prolonged differentiation, making these models poorly suited to the throughput and turnaround times required for large-scale drug screening [55,68]. 3D organoid platforms could help address this limitation.

### 3.3. Advanced Human Models: Organoids and Ex Vivo Brain Slices

Recent research has developed three-dimensional (3D) cerebral organoid systems from stem cells (Figure 1, Panel 2). Unlike 2D monolayers, these systems allow stem cells to organise into complex, layered structures that resemble the architecture of the developing human brain [69,70]. They also exhibit a wide range of cell types and can sustain spontaneous neural network activity for long periods in culture. These features make them useful as models for studying neurodegenerative diseases [70].

In the context of regulating MAPT exon 10 in 3D organoids, the researchers observed that the 3D cerebral organoid microenvironment accelerates neuronal maturation and induces MAPT exon 10 alternative splicing more rapidly than 2D cultures [68].

After 16–24 weeks of growth, 3D wild-type human organoids demonstrate robust endogenous expression of all six adult tau isoforms. These expression levels are the physiological 3R/4R ratio required for functional studies [71]. When 3D organoids with FTDP-17 mutations are studied, accelerated maturation profiles reveal early pathological phenotypes that remain undetectable in 2D monolayers [55,68,71]. For example, in 3D cerebral organoids harbouring the Tau-V337M mutation—which, like P301L, affects tau protein aggregation rather than MAPT exon 10 splicing regulatory elements- splicing dysregulation of unrelated synaptic transcripts and defects in glutamatergic signalling (including altered expression of the RNA-binding protein ELAVL4) occur before neuronal loss [68]. Notably, these splicing alterations are a downstream consequence of tau proteotoxicity rather than a direct alteration of MAPT exon 10 splicing by the V337M mutation [68]. In 3D cortical organoids harbouring the intronic *MAPT* IVS10+16 mutation, the accelerated developmental trajectory manifests as disrupted cortical differentiation and synaptic maturation alongside mitochondrial dysfunction and pathological tau accumulation [72].

This rapid unmasking of mutation-specific phenotypes triggers a cascade of downstream neurodegenerative changes that are difficult to replicate in 2D. It is indeed possible to observe accelerated tau hyperphosphorylation [72], mitochondrial dysfunction with marked structural alterations in mitochondrial networks [30,72], synaptic loss and network failure [30,72], and progressive tau aggregation, which more closely mirrors the ageing human brain [44,71,72].

Therefore, 3D brain organoids are well suited for studying how basic RNA biology relates to preclinical drug discovery. They offer a human-specific way to test the long-term effectiveness and safety of molecular therapies that aim to correct alternative splicing. A complementary but distinct human tissue-based platform is organotypic brain slice cultures (Figure 1, Panel 2). These are generated from neocortical tissue during surgery, typically peritumoral or epilepsy-surgery access tissue [26,27], and preserve the fully differentiated cytoarchitecture and cell-type diversity of the adult human brain. They also preserve pre-existing synaptic connectivity and the mature, *in vivo*-like 3R/4R tau splicing profile at the time of resection. The organotypic cultures represent a valuable intermediate model for testing the acute pharmacodynamic effects of candidate splicing-corrective ASOs, SMCs, or RNP complexes in adult human neural tissue. They could cover the gap between cellular and organoid platforms and *in vivo* animal studies, before a candidate advances to clinical testing [27].

To date, the use of human organotypic slice platforms to study FTDP-17 is growing [73]. Hybrid iPSC-organoid slice cultures have revealed early pharmacologically targetable astroglial and neuronal pathology in FTLD-spectrum diseases caused by C9ORF72 mutations. These data demonstrate the feasibility of using this platform for drug-discovery studies in FTD [74].

Although this model does not involve MAPT exon 10 splicing itself, *ex vivo* organotypic brain slice cultures open the possibility of screening candidate exon 10 splicing molecules in FTDP-17 patient-derived neural tissue as such biobank resources become more available [75].

### 3.4. In Vivo Animal Models for MAPT Exon 10 Splicing

*In vivo* models play a key role in testing how well splicing-corrective therapies work and whether they can be used in real-world settings (Figure 1, Panel 3). These models help researchers study how treatments spread through the brain, cross the Blood–Brain Barrier, behave in the body over time, remain safe in the long term, and reverse disease-related problems with thinking and movement [25,76,77].

Still, modelling MAPT exon 10 alternative splicing in rodents is especially challenging because of evolutionary differences, so more advanced genetic engineering is needed. Early *in vivo* mouse models addressed the problem of expressing 4R tau in normal rodents, which naturally include exon 10 [1,25], by using transgenic mice that overexpress mutant human *MAPT* cDNAs. The JNPL3 and rTg4510 P301L tau mouse lines exhibit key features of tau disease, including hyperphosphorylation, neurofibrillary tangles, movement impairments, and cortical shrinkage [78,79]. However, these models use cDNA, so they skip the natural MAPT alternative splicing process and rely on high levels of a single tau isoform. This modification limits their ability to mimic the splicing-related disease process in human FTDP-17.

To address this issue, researchers developed the humanised tau (htau) mouse by knocking out the endogenous mouse tau gene (mTau^−/−^) and introducing the entire human *MAPT* genomic locus within a bacterial or yeast artificial chromosome (BAC/YAC) [25]. Adult htau mice express all six human tau isoforms and exhibit a human-like alternative splicing profile, with a 1:1 ratio of 4R and 3R isoforms, albeit with a modest preference toward 3R tau accumulation over time [25].

As reported in the literature, this mouse model provides an *in vivo* platform for testing the pharmacokinetic delivery, safety and exon-skipping efficiency of human-targeted ASOs [76,77]. Furthermore, the htau mice provided direct causal evidence that a shifted 4R/3R ratio, independent of a genetic FTDP-17 mutation, is itself sufficient to drive pathological changes [80].

To model the pathological exon 10 inclusion characteristic of FTDP-17, scientists developed transgenic mouse models carrying human MAPT splicing mutations. Among these, the Tau-N279K and Tau-IVS10+16 models reproduce mutation-driven alterations in exon 10 splicing *in vivo*, providing systems to investigate the 3R/4R tau ratio and evaluate splicing-corrective therapies [77,81].

The Tau-N279K model harbours the exonic N279K mutation, which promotes exon 10 inclusion and increases 4R tau production [81]. These mice develop progressive tau hyperphosphorylation and neuropathological abnormalities accompanied by behavioural and motor deficits, providing *in vivo* evidence linking mutation-driven splicing dysregulation to tau pathology [81]. Notably, an earlier N279K transgenic model showed mutation-dependent cognitive deficits in the absence of neurofibrillary tangle formation, indicating that functional impairment may precede overt tangle pathology [82].

Similarly, the Tau-IVS10+16 model includes the intronic IVS10+16 C>T mutation, which destabilises the exon 10–intron 10 stem-loop and increases exon 10 inclusion. This results in progressive accumulation of 4R tau in the brain and a gradual decline in cognitive function, similar to the neurodegeneration observed in patients [77]. Together, these models provide important *in vivo* evidence that FTDP-17-associated mutations drive disease by disrupting splicing regulation of MAPT exon 10.

## 4. Therapeutic Strategies to Correct MAPT Exon 10 Splicing

**Figure 2:**
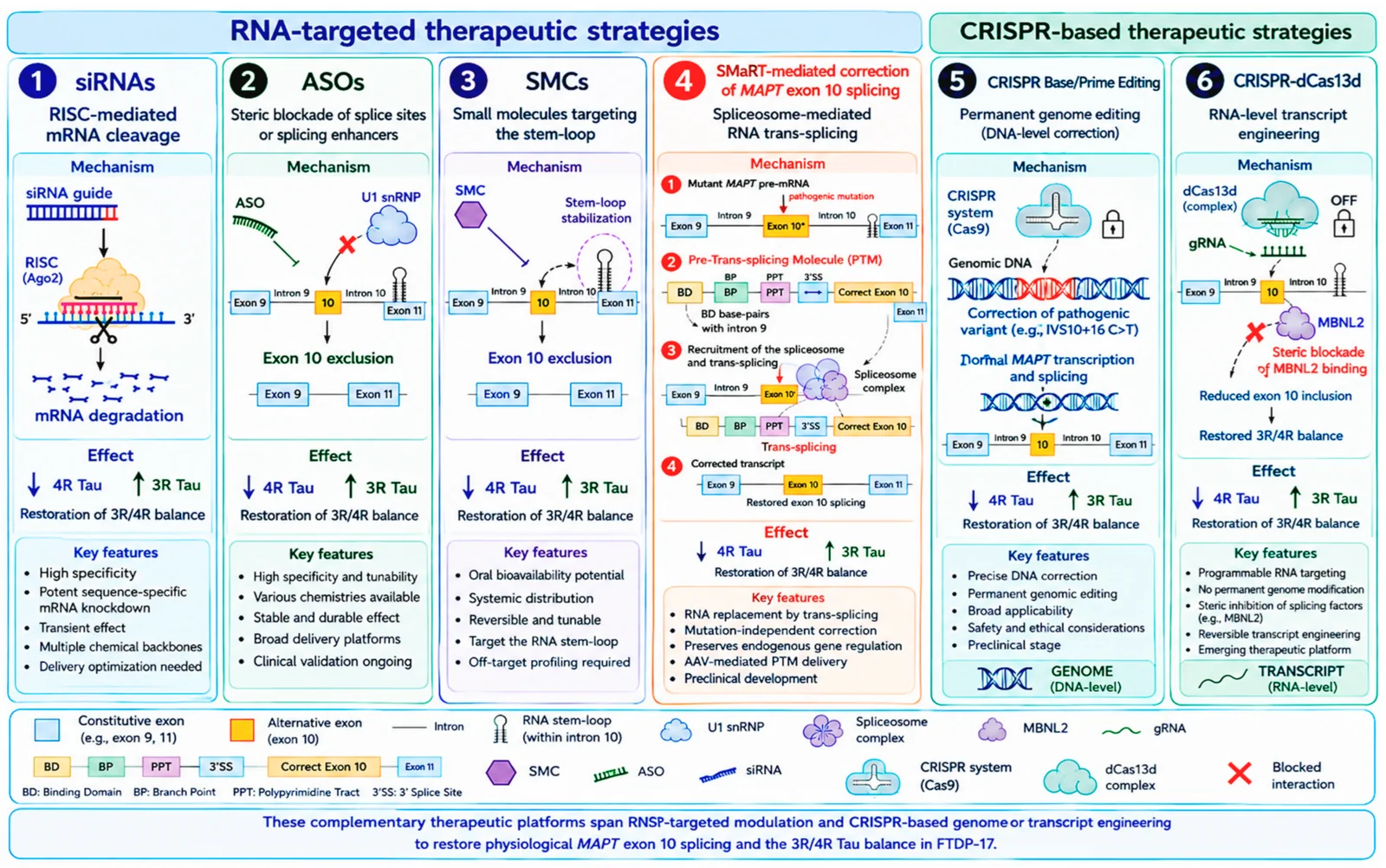
Schematic representation of therapeutic strategies targeting splicing of MAPT exon 10. (1) siRNAs for selective reduction of 4R-tau mRNA, (2) ASOs for modulation of exon 10 splicing, (3) Small-Molecule Compounds targeting regulatory RNA structures (SMCs), (4) SMaRT for RNA *trans*-splicing, (5) CRISPR base/prime editing for permanent correction of pathogenic genomic variants, and (6) CRISPR-dCas13d for programmable RNA-level modulation of exon 10 splicing without genomic modification. Together, these strategies restore the physiological 3R/4R tau ratio.

The main goal of therapeutic approaches in FTDP-17 is to restore the 1:1 ratio of 3R and 4R tau isoforms [15]. Since both intronic and exonic alternative splicing mutations can cause aberrant exon 10 inclusion, current strategies focus on regulating exon 10 splicing at various levels, from direct control of *MAPT* pre-mRNA splicing to programmable RNA transcript editing and permanent genome editing [83,84,85,86]. Figure 2 provides a schematic overview of the complementary strategies discussed in the following sections [1,24,83,84,85,86,87,88].

### 4.1. RNA Interference and Small Interfering RNA (siRNA)

RNA-lowering strategy exploits the endogenous RNA interference (RNAi) pathway, first described in 1998 [89]. It uses Small Interfering RNAs (siRNAs), short double-stranded RNA duplexes that cleave the target mRNA sequence post-transcriptionally instead of correcting the splicing event (Figure 2, Panel 1). They have since matured into a well-established therapeutic modality [90]. In FTDP-17 cases driven by exon 10 splicing mutations, siRNA-based knockdown strategies can lower total tau levels and reduce tau protein aggregation [91].

More broadly, complementary siRNA-based strategies for allele-specific silencing have also been explored [92,93], including selective silencing of individual mutant alleles or exonic sequences, providing a broader conceptual framework for developing exon 10-directed siRNA strategies against MAPT.

However, this isoform-selective strategy remains a patent-based approach rather than a quantitatively validated preclinical demonstration, whereas the currently available MAPT siRNA studies have primarily focused on total tau reduction. Indeed, a patent has been granted for an exon 10-specific siRNA silencing strategy for the treatment of tauopathies, including FTDP-17, demonstrating its potential to reduce aberrant 4R tau expression and restore the physiological 3R/4R ratio [94]. The clinical use of siRNA-based drugs underscores their therapeutic potential. Several siRNA drugs have already been approved by the U.S. Food and Drug Administration (FDA) to target liver or other peripheral tissues [95,96,97,98,99,100]. These approvals show that chemically optimised siRNAs can achieve potent, durable, and clinically tolerated gene silencing in humans [100,101], setting an important precedent for extending RNAi-based approaches to Central Nervous System (CNS) disorders [102].

Preclinical studies further demonstrate that targeting MAPT mRNA with siRNAs is feasible. Xu et al. [91] found that an Accell SMARTpool siRNA targeting MAPT reduced tau levels in cultured neurons; when delivered directly to the brain, it reduced toxic tau levels in the hippocampus of P301S transgenic mice without evidence of neurotoxicity, neuroinflammation, or cell death [91].

In primary cortical neurons from P301S mice, exposure to 250 nM MAPT siRNA for 96 h reduced human MAPT mRNA by approximately 60% and tau protein by approximately 40%, demonstrating concentration-dependent target engagement at the cellular level [91]. *In vivo*, a single 0.4-nmol intrahippocampal administration reduced AT180 immunoreactivity, as assessed by optical density measurement, to approximately 54.7% of control levels at 14 days, corresponding to a reduction of approximately 45%; however, this effect was no longer detectable at 21 or 28 days, indicating limited durability after local CNS administration [91]. Notably, intracerebroventricular administration of the same siRNA did not produce detectable tau knockdown, further highlighting the importance of local parenchymal delivery for this approach [91]. Researchers have also tested allele-specific siRNA for FTDP-17: Miller et al. [93] designed siRNAs targeting several MAPT mutations and identified one directed against the V337M mutation capable of selectively reducing the mutant tau allele in cell models, demonstrating that siRNAs can discriminate between mutant and normal MAPT transcripts differing by only a single nucleotide [93]. In a heterozygous cellular model, the optimised V337M-directed siRNA (siA9C12) reduced mutant tau to approximately 16.7% of control levels, compared with 12.7% in non-heterozygous transfections, while producing minimal alteration of wild-type tau expression [92,93]. Importantly, however, residual silencing of the wild-type allele was still detectable, indicating that complete allele specificity was not achieved [92,93].

Together, these results support both general MAPT silencing and mutation-selective RNAi as candidate treatments for FTDP-17. However, these approaches still require substantial improvement for use in the brain, particularly for RNA delivery [102].

Overall, the available evidence indicates that MAPT-targeting siRNAs can achieve substantial target knockdown, with approximately 40–60% reduction in tau or MAPT mRNA in cellular and local *in vivo* models, but the demonstrated effects remain primarily focused on total tau lowering rather than correction of the 3R/4R isoform ratio. Moreover, the limited persistence observed after a single local CNS administration highlights CNS delivery and durability as major translational challenges.

### 4.2. Antisense Oligonucleotides (ASOs)

Antisense oligonucleotides (ASOs) are a promising approach for correcting MAPT exon 10 splicing defects. ASOs are short (typically 15–25 nucleotides) single-stranded synthetic nucleic acid sequences designed to bind target pre-mRNA via Watson–Crick base pairing. Unlike gapmer ASOs, which recruit RNase H to degrade the target transcript, splice-modulating ASOs are chemically modified to resist degradation. They act as steric blockers, preventing *cis*-acting regulatory sequences (splice donor/acceptor sites or exonic/intronic splicing enhancers) from being recognised by the spliceosomal machinery (Figure 2, Panel 2) [86].

In FTDP-17, ASOs are engineered to correct the pathogenic overproduction of 4R tau by promoting exon 10 skipping, restoring the physiological 1:1 ratio. Early proof-of-concept studies in *MAPT* minigenes and immortalised cell lines showed that targeting the 5′ splice site of exon 10 shifts splicing, promoting exon 10 skipping.

This strategy was validated in patient-derived induced pluripotent stem cell (iPSC) neurons carrying the IVS10+16 or N279K mutations. In these neurons, treatment restored the splicing imbalance, reduced tau hyperphosphorylation, and recovered normal retrograde axonal transport [88,103,104].

*In vivo*, preclinical studies in humanised hTau and genomic-replacement mouse models showed that a single intracerebroventricular (ICV) injection of a splice-switching ASO can achieve wide distribution throughout the CNS, entry into neurons and glia, and sustain a corrected 3R/4R ratio for several months, resulting in reduction in tau aggregation, halting of cortical atrophy, and reversal of cognitive and motor deficits [103]. Next-generation platforms utilising 2′-O,4′-C-ethylene-bridged nucleic acid (ENA-ASO) chemistry have achieved exceptional intracellular stability and nuclease resistance, with a single administration restoring the 3R/4R ratio in humanised hTau mice for several months without any systemic toxicity [103].

Researchers later applied this method to a FUS/SFPQ-silenced humanised tau mouse model with exon 10 hyper-inclusion. A single intracerebroventricular dose of a long-acting ENA-modified ASO corrected the abnormal 3R/4R ratio in both the mouse brain and patient-derived hiPSC neurons [103]. The ASO remained in the brain for about six months and maintained splicing correction for up to two years, which is longer than a similar 2′-O-methoxyethyl (MOE)-modified ASO, while keeping total MAPT expression unchanged [103]. Importantly, a single 50-μg ICV dose of the ENA-modified ASO EN-06 resulted in approximately 500–700 pmol/g of compound in hippocampal tissue at 6 weeks, providing a quantitative measure of CNS exposure [103]. This prolonged pharmacodynamic effect likely reflects two complementary factors: the intrinsic biostability of ENA chemistry, including high nuclease resistance and increased affinity for complementary RNA, which sustains ASO stability and target engagement; and the steric-blocking mechanism of splice-modulating ASOs, which requires stoichiometric occupancy of the target pre-mRNA rather than catalytic turnover. Residual, sub-quantifiable ASO concentrations retained within the nuclei of post-mitotic neurons may therefore sustain exon 10 skipping well beyond the detectable tissue persistence of the compound [103,105]. Antisense strategies have also been used to reduce total tau rather than selectively correct the 3R/4R ratio. Gapmer ASOs targeting human tau reduce tau pathology and seeding activity, increase survival rates, and show broad CNS distribution in preclinical models [76,80]. Similarly, morpholino oligomers designed to induce skipping of constitutive MAPT exons reduce tau mRNA and protein levels in neuroblastoma cells and human MAPT transgenic mice [106]. Together, these studies highlight the versatility of antisense approaches, which can selectively correct exon 10 alternative splicing or reduce overall tau expression.

The clinical translatability of ASO-based tau modulation has been further underscored by the success of other splice-switching ASOs in medicine, such as nusinersen for spinal muscular atrophy (SMA) [87], and by the completed Phase 1b trial of MAPTRx (ISIS 814907/BIIB080), now developed as diranersen.

This application demonstrated that intrathecal administration of a tau-targeting ASO was well tolerated and produced dose-dependent reductions in CSF total tau in patients with mild Alzheimer’s disease [88,104,107], a peer-reviewed proof-of-concept for human CNS tau-ASO pharmacology that directly informed the subsequent Phase 2 CELIA trial [107]. In the multiple-ascending-dose study, mean CSF total tau reductions at 8 weeks after the last dose were 30%, 40%, 49%, and 42% in the 10 mg, 30 mg and 60 mg monthly and 115 mg quarterly cohorts, respectively. In the two higher-dose cohorts, reductions reached 56% and 51% for CSF total tau and 56% and 46% for p-tau181, respectively, at 24 weeks after the last dose, and were maintained with quarterly dosing [104].

Moreover, exploratory tau-PET analyses showed reduced tau accumulation versus placebo at week 25. In contrast, at week 100 tau-PET signal was reduced from baseline across all assessed regions, with the largest reduction observed in the temporal composite (−0.71 SUVR) [104].

Taken together, the convergence of mechanistic diversity, preclinical validation, and early clinical proof-of-concept provides renewed optimism for the broader ASO-based therapeutic landscape in tauopathies [108]. Collectively, these findings highlight ASOs as one of the most advanced therapeutic strategies for MAPT modulation, while also underscoring the distinction between isoform-selective splice correction and non-selective tau lowering.

### 4.3. Small-Molecule Splicing Modulators (SMCs)

Unlike splice-modulating ASOs, which require repeated invasive administration due to limited ability to cross the blood–brain barrier (BBB) [86], small-molecule splicing modulators (SMCs) offer potential for oral administration and broader CNS distribution, including access to deep brain regions (Figure 2, Panel 3) [44]. Their development for FTDP-17 aims to reduce aberrant MAPT exon 10 inclusion and restore the physiological 3R/4R tau balance. Identified compounds act mainly by binding regulatory RNA structures, such as the MAPT intron 10 stem-loop, or by modulating splicing factor interactions with these elements [44,109]. Structure-guided and hybrid RNA-targeting approaches have also been explored to enhance recognition of structured MAPT RNA targets [110,111].

The overall discovery and screening strategies for MAPT-targeting SMCs have been reviewed elsewhere [111].

Preclinical studies provide evidence for efficacy in humanised models.

Structure-activity optimisation of the parent compound BPN-15477 identified four lead SMCs (PTC-553, PTC-700, PTC-775, and PTC-003), which induced a twofold increase in 3R MAPT transcript (EC2x) at nanomolar concentrations (4.9–20.9 nM) in a HEK293T-*MAPT* minigene assay [44]. In an orthogonal assay measuring endogenous 3R tau protein, EC1.3x values ranged from 7.4 to 175.5 nM, with PTC-003 and PTC-775 showing the highest potency [44]. In patient-derived iPSC neurons, prolonged treatment further enhanced SMC activity. After 3 weeks, PTC-700, PTC-775, and PTC-003 reduced 4R MAPT mRNA with EC50 values ranging from 0.7 to 21.3 nM in P301L neurons and from 1.1 nM to 159.5 nM in S305N neurons, with reductions exceeding 80% at higher concentrations for PTC-775 and PTC-003 [44]. At the protein level, PTC-775 produced approximately 90% maximal reduction of 4R tau in P301L neurons, with EC50 values of 5.6–28.4 nM, whereas PTC-700 and PTC-003 showed EC50 values of approximately 57.1 nM and 30.2–83.6 nM, respectively [44]. In S305N neurons, PTC-700 and PTC-775 reduced 4R tau by approximately 60–80%, with EC50 values of 46.6–75.8 nM, while PTC-003 achieved a similar maximal reduction with higher EC50 values of 166.6–173.0 nM [44].

Notably, P301L affects tau protein aggregation rather than MAPT exon 10 splicing regulatory elements, whereas S305N is a genuine exon 10 splicing mutation (Table 1); the use of these two mutant contexts allowed the activity of splicing-modulatory compounds to be evaluated across distinct disease-relevant cellular models, although only S305N directly models an exon 10 splicing defect [44]. SMC treatment also reduced hyperphosphorylated tau and other pathological tau species. After 3 weeks, PTC-700 and PTC-775 reduced total tau and multiple pTau species in P301L neurons by approximately 90–100%, while PTC-700 and PTC-775 nearly eliminated pTau S396 oligomers at concentrations above 1 μM, whereas PTC-003 produced an approximately 80% reduction at 1 nM with prolonged treatment [44]. PTC-775 also reduced insoluble total tau and oligomeric pTau S396 by more than 80% in P301L neurons and by more than 90% in S305N neurons [44]. These effects were accompanied by rescue of neuronal viability under glutamate-induced stress, with viability increasing from approximately 20–30% in vehicle-treated P301L neurons to approximately 90% following treatment with 1 μM SMC [44].

After oral administration (with cotreatment using the cytochrome P450 inhibitor 1-aminobenzotriazole to sustain systemic exposure) in a humanised gene-replacement *MAPT*-N279K mouse model, PTC-003 produced a dose-dependent increase in the 3R/total tau ratio and a corresponding reduction in pathogenic pTau T231, both reaching statistical significance at 2.5 mg/kg (*p* = 0.007 and *p* = 0.009, respectively), without effects on total tau levels or body weight [44]. A single oral dose of PTC-003 (7 mg/kg) increased the brain 3R/4R MAPT mRNA ratio, with the maximal effect observed 16 h after dosing [44]. An independent SMC series similarly stabilised the MAPT exon 10–intron 10 stem-loop.

In a complementary RNA-targeted SMC study, treatment with the optimised compound at 1.5 μM reduced the 4R/3R MAPT mRNA ratio by approximately 42% in cells carrying the DDPAC *MAPT* mutation, compared with vehicle-treated cells [112]. 

In a humanised htau transgenic mouse model, it reduced the 4R/3R mRNA ratio and cortical phosphorylated tau while improving behavioural deficits, supporting brain penetration and efficacy after oral administration [112]. *In vivo*, oral administration of the same compound at 100 mg/kg every other day for 20 days reduced the brain 4R/3R MAPT mRNA ratio by approximately 38%, from 0.91 to 0.56, and decreased exon 10 inclusion (PSI10) from 47% to 36%, while total human MAPT transcript levels remained unchanged [112]. However, these studies assessed target engagement during active treatment, and the persistence of splice correction after treatment cessation has not yet been systematically established for MAPT-targeting SMCs. The clinical potential of this therapeutic class is supported by the approval of the orally administered splicing modulator risdiplam for spinal muscular atrophy, demonstrating that small molecules can correct pre-mRNA splicing in humans [113,114,115].

For MAPT-targeting SMCs, transcriptome-wide RNA sequencing showed that PTC-700 altered splicing in only 0.79% of expressed exon triplets (933 of 117,447) at a concentration matching its 3R MAPT EC2x, a selectivity profile comparable to that of BPN-15477 but achieved at approximately 1000-fold lower concentration [44]. Nonetheless, emerging computational approaches may improve compound selectivity [116]. Additional preclinical evidence from other splicing disorders further supports the potential of orally bioavailable SMCs to achieve meaningful phenotypic rescue, providing a translational precedent for their development in FTDP-17 [117,118,119].

### 4.4. Spliceosome-Mediated RNA Trans-Splicing (SMaRT)

Spliceosome-mediated RNA *trans*-splicing (SMaRT) repairs mutated pre-mRNA transcripts after transcription. Instead of producing extra proteins, it uses the cell’s spliceosome to join an introduced pre-*trans*-splicing RNA molecule (PTM) to the target RNA (Figure 2, Panel 4). The PTM includes a binding domain that matches the intron, a branchpoint/splicing domain, and a wild-type coding sequence, such as a corrected exon 10–12 cassette, which replaces the mutated transcript [120,121].

For FTDP-17, PTMs have been designed either to carry a 3′ splice site and a downstream (3′) wild-type coding sequence, *trans*-splicing onto the endogenous 5′ splice site of *MAPT* intron 9 to convert E10^−^ to E10^+^ transcripts [120], or to carry a 5′ splice site and an upstream (5′) coding sequence, trans-splicing onto the endogenous 3′ splice site of intron 10 to promote exon 10 exclusion [122]. This SMaRT are delivered into cells using viral vectors, such as lentivirus or AAV [123]. In each case, the spliceosome is guided to incorporate the introduced wild-type sequence in place of the mutated transcript segment. Initial studies in HEK293 cells and primary neurons showed this method reduced mutated transcripts and restored the 3R/4R tau isoform ratio [120,122]. In the initial proof-of-concept study, conversion of exon 10-negative to exon 10-positive MAPT transcripts was achieved with approximately 34% efficiency in the most effective PTM configuration [120]. In a subsequent study addressing FTDP-17-associated splicing defects, trans-splicing efficiencies of approximately 11% for the DDPAC+14 mutation and 7% for the N279K mutation were reported, demonstrating that correction efficiency can vary substantially according to the pathogenic splicing context [122]. Later, *in live* mice, delivering a PTM targeting intron 9 with a lentivirus corrected the 3R/4R ratio in the brain [123]. In cultured cortical neurons from htau mice, lentiviral delivery of the intron 9-targeting PTM produced a dose-dependent increase in the 4R/3R ratio, from 0.1 in control neurons to 0.4 and 0.6 at multiplicities of infection (MOIs) of 5 and 10, respectively; at MOI 10, this corresponded to approximately 36% conversion of tau isoforms [123].

Further research showed that correcting isoforms with SMaRT improved cognitive and motor impairments in a preclinical tauopathy model [123]. In a humanised htau mouse model, intrastriatal delivery of PTM4R significantly increased the 4R/3R ratio (>1) compared with 3R-predominant htau controls (4R/3R < 1), without altering total tau levels [124]. In the same study, PTM4R was administered to 6-month-old htau mice and animals were evaluated at 12 months, demonstrating sustained isoform modulation over the 6-month observation period together with rescue of cognitive or motor deficits depending on the brain region targeted [124].

SMaRT’s main benefits are its precise sequence targeting and preservation of the natural MAPT promoter controlling the repaired transcript. However, using SMaRT in treatments is challenging. *Trans*-splicing is less efficient than *cis*-splicing and delivering the PTM requires high doses of AAV vectors, which can cause immune reactions and inflammation. Overall, the available studies provide quantitative evidence of partial splice correction, with *trans*-splicing efficiencies ranging from approximately 7% to 36% across cellular models and experimental configurations [120,122,123]. However, these values are not directly comparable to standardised potency measures such as EC50, and the available *in vivo* studies have not systematically evaluated persistence of splice correction after cessation of PTM expression.

Researchers are working to improve PTM design and AAV types to address these problems.

### 4.5. CRISPR-Based Approaches: RNA and DNA-Level

CRISPR technologies offer two complementary strategies for correcting MAPT exon 10 alternative splicing: reversible RNA-level transcript engineering, whose duration of effect depends on sustained CNS delivery, and permanent DNA-level correction, which could achieve durable effects after a single intervention but requires rigorous control of specificity and long-term safety given its irreversible nature.

#### 4.5.1. RNA-Level CRISPR: dCas13d Transcript Engineering

CRISPR-dCas13d transcript engineering uses a Cas13d variant that does not cleave RNA, guided by specific gRNAs, to control RNA without altering the DNA [24]. When targeted to regulatory elements near exon 10, such as MBNL-binding sites, dCas13d could interfere with the splicing regulator activity and promote exon 10 exclusion. This method offers a reversible way to restore normal splicing [24]. It exploits the same regulatory features that control exon 10 inclusion during development. Importantly, MAPT-specific proof-of-concept data are now available. Recinos et al. [24] demonstrated that simultaneous targeting of the distal intronic MBNL-binding site with four gRNAs reduced exon 10 inclusion from approximately 60% to 27% in a *MAPT* minigene system, corresponding to an approximately 55% relative reduction in exon 10 inclusion. These findings provide direct evidence that steric targeting of an intronic MBNL-binding site can modulate MAPT exon 10 splicing and alter the predicted tau isoform balance [24].

#### 4.5.2. DNA-Level CRISPR: Base and Prime Editing

Base and prime editing, on the other hand, are designed to permanently correct harmful *MAPT* mutations in DNA without making double-stranded breaks or needing donor DNA templates [84,85]. These methods could potentially correct intronic mutations, such as IVS10+16 C>T, that disrupt the exon 10–intron 10 stem-loop, helping restore the normal regulatory sequence and balance between 3R/4R tau.

The choice of CRISPR-based strategy depends on the specific nucleotide change and the required corrective substitution: some transition mutations may be amenable to Cytosine or Adenine Base Editing, depending on the direction of the correction and the editing context [84,125], whereas other transition mutations, transversions, and small deletions may be more appropriately addressed by Prime Editing [85]. Some variants may therefore be potentially addressable by more than one editing strategy. Table 1 summarises the classification of FTDP-17 mutations by nucleotide change and potential compatibility with these editing approaches.

Potential editing strategies are inferred from the nucleotide change required to restore the reference sequence and the canonical editing activities of CBEs and ABEs. Actual editability would additionally depend on PAM availability, target-strand orientation, editing-window constraints, bystander editing, and locus-specific activity; therefore, these assignments represent theoretical compatibility rather than experimentally validated correction of the corresponding MAPT variants.

While base and prime editing have been used to fix disease-related variants in human iPSC models for other genetic diseases, no published study yet shows their use for *MAPT* exon10/intron 10 mutations. For context, systematically optimised prime editing achieved correction efficiencies of approximately 58% for the CFTR F508del mutation in immortalised airway epithelial cells and approximately 25% in patient-derived airway epithelial cells [126]. In a separate study, prime editing corrected the RBM20-P633L mutation in approximately 35% of homozygous human iPSC-derived cardiomyocytes, with restoration of RBM20 nuclear localisation and normalisation of cardiac CAMK2D splicing [127].

These studies provide proof-of-concept that substantial prime-editing efficiencies can be achieved in human disease-relevant cells, including post-mitotic cardiomyocytes, but editing efficiency is highly locus- and variant-dependent and therefore cannot be directly extrapolated to pathogenic MAPT variants.

Importantly, recent, *in vivo*, studies further support the feasibility of DNA editing in the central nervous system. Optimised dual-AAV delivery of prime editors achieved editing efficiencies of up to 42% in the cortex of adult mice [128], demonstrating that efficient prime-editor delivery and activity can be achieved in the mammalian brain. In a more directly analogous example, CRISPR base editing delivered to the striatum of Huntington’s disease mouse model disrupted a splice-acceptor site in the HTT gene, achieving an exon-skipping rate of approximately 9.1% in bulk striatal tissue and reducing mutant HTT fragment formation, aggregation, functional deficits, and brain atrophy *in vivo* [129]. This splice-site-directed strategy provides a relevant conceptual precedent for targeting pathogenic splicing events and illustrates that therapeutic outcomes depend on the specific genomic target and editing design. Using these methods in the adult central nervous system remains difficult due to challenges with delivery, the size of the editing tools, and the need to avoid long-term off-target effects [130]. Researchers are exploring non-viral delivery methods, such as lipid nanoparticles (LNPs) and transiently engineered ribonucleoprotein (RNP) complexes, to facilitate future genome-editing approaches for FTDP-17 [130].

Overall, RNA-level CRISPR approaches already provide quantitative evidence of MAPT exon 10 splice modulation, whereas DNA-level editing remains an earlier-stage strategy for FTDP-17, with no MAPT-specific evidence yet defining *in vivo* editing efficiency, therapeutic efficacy, or long-term durability.

### 4.6. Structure-Specific RNA Stem-Loop Binders

Structure-specific RNA binders represent an emerging approach to directly stabilise the MAPT exon 10–intron 10 stem-loop, which is disrupted by pathogenic intronic mutations such as IVS10+14 and IVS10+16. Aptamers and other conformation-selective compounds can bind the mutant RNA structure and potentially restore its ability to restrict access to the 5′ splice site, reducing aberrant exon 10 inclusion [109,131].

An early proof-of-concept small molecule, compound 2, bound the DDPAC (C+14U) mutant stem-loop with a dissociation constant (Kd) of approximately 10 μM (compared with 14 μM for the wild-type stem-loop, and no detectable binding to the DDPAC+I17T mutant, Kd > 100 μM) and achieved statistically significant modulation of the 4R/3R splicing ratio at 60 μM in a cellular *MAPT* minigene model, illustrating the feasibility of this approach albeit at a substantially higher concentration than more recently optimised SMCs (Section 4.3) [109].

Selectivity was further supported by the absence of any significant effect on wild-type MAPT splicing or on an unrelated endogenously expressed transcript, CAMKK2 (calcium/calmodulin-dependent protein kinase kinase 2), at the same concentration [109]. An EC50 or maximal splice-correction value was not reported, and the compound was not evaluated *in vivo* [109]. Structure-based studies have shown that the RNA major groove of this stem-loop can be specifically recognised by aminoglycoside antibiotics such as neomycin, providing a structural basis for rational small-molecule design targeting this element [132]. Neomycin bound both wild-type and mutant MAPT stem-loop sequences with micromolar affinity and increased their thermal stability, with ΔTm values of approximately 8–12 °C; however, the destabilised +3 and +14 mutant stem-loops remained significantly less stable than the wild-type RNA even in the presence of neomycin, indicating only partial structural rescue [132]. Functionally, direct stabilisation of the stem-loop, achieved experimentally through sequence variants that increased its melting temperature by 2.3–5.7 °C, correlated with a significant reduction in the 4R/3R tau mRNA ratio (r^2^ = 0.75, *p* < 0.05), supporting a direct relationship between stem-loop stability and exon 10 splicing outcome [131].

This strategy remains at an early preclinical stage compared with ASOs and SMCs but offers a distinct structure-based approach for mutation-associated splicing defects. An additional advantage of aptamers is their potential for non-invasive CNS delivery through receptor-mediated BBB transcytosis, including approaches targeting transferrin receptor or LRP1 [133]. However, such receptor-mediated delivery strategies remain largely preclinical, and challenges including nuclease degradation, systemic clearance, BBB heterogeneity, and intracellular trafficking limit their current translational maturity [133].

Other RNA aptamers have been developed to target pathological tau protein aggregation rather than MAPT RNA.

Notably, the DNA aptamer BW1c binds tau protein with high affinity (Kd = 6.6 nM) and effectively inhibits arachidonic acid-induced tau oligomerisation and aggregation [134]. In addition, retro-orbital administration demonstrated that BW1c can cross the blood–brain barrier and reach neuronal cell bodies [134]. By acting at the protein level, these approaches provide a complementary downstream strategy to limit the aggregation propensity of residual pathogenic 4R tau [134,135].

In contrast, upstream RNA- and DNA-targeting strategies aim to restore the physiological 3R/4R ratio [120,121,122,123,124].

Taken together, the available quantitative evidence highlights substantial differences in potency, efficacy, durability, and translational maturity among the therapeutic approaches discussed. Direct cross-platform comparisons remain challenging because the studies use different experimental models, delivery routes, doses, and efficacy measures.

Nevertheless, ASOs currently provide the most extensive evidence for direct MAPT exon 10 splice correction, including restoration of the 3R/4R ratio and prolonged *in vivo* activity after a single CNS administration [103], while clinical MAPTRx/diranersen studies provide human proof-of-concept for CNS tau lowering rather than direct splice correction [104,107].

SMCs combine nanomolar potency in several cellular assays with measurable modulation of MAPT exon 10 splicing and pathological tau following oral administration in humanised models [44,112], although the persistence of splice correction after treatment cessation remains insufficiently characterised. siRNA approaches can achieve substantial MAPT/tau knockdown but have primarily demonstrated total tau reduction and limited durability following local CNS administration [91,93].

SMaRT has demonstrated partial splice correction in cellular and animal models, whereas CRISPR-based approaches remain at an earlier stage, with quantitative MAPT-specific evidence currently available mainly for RNA-level splice modulation [24]. Structure-specific RNA binders also remain at an early preclinical stage, with quantitative evidence currently limited to RNA structural stabilisation, binding affinity, and modulation of exon 10 splicing in cellular models, while *in vivo* therapeutic efficacy has not yet been established [109,131,132].

Overall, these findings indicate that the therapeutic approaches differ not only in their reported magnitude of effect but also in the biological endpoint targeted, CNS delivery requirements, durability, and level of preclinical or clinical validation, which are critical considerations when assessing their translational potential.

Table 2 summarises the main therapeutic strategies targeting MAPT exon 10 splicing, their mechanisms, validated experimental models, advantages, and current limitations.

## 5. Preclinical Pharmacology: ASOs vs SMCs for MAPT Exon 10 Alternative Splicing

To develop effective exon 10 splicing-corrective therapies, it is important to consider the pharmacological, pharmacokinetic, and biophysical properties of antisense oligonucleotides (ASOs) and small-molecule splicing modulators (SMCs) [44,105,136].

Their physicochemical properties result in distinct profiles in CNS delivery, tissue distribution, persistence, dosing, and target specificity. These differences arise from chemical improvements, starting with early unmodified oligonucleotides and progressing to chemically stabilised ASOs, and optimised small molecules now studied for therapy [137]. The physical dimensions and chemical properties of ASOs and SMCs result in marked differences in their pharmacological profiles across all preclinical parameters relevant to CNS drug development [86,138].

ASOs are large (~7–10 kDa), highly hydrophilic and negatively charged macromolecules that exhibit negligible passive diffusion across the blood–brain barrier (BBB).

These molecules require direct, invasive administration into the central nervous system (CNS) via intrathecal (IT) or intracerebroventricular (ICV) injection. After administration, they distribute via cerebrospinal fluid (CSF) flow, diffusing into cortical and hippocampal regions and, more slowly, into subcortical structures [105]. Modern ENA- or MOE-chemistry ASOs resist nuclease degradation and have an intracellular half-life of several months [103,105], allowing long intermittent dosing.

By contrast, SMCs are small (~300–500 Da), uncharged, lipophilic molecules that can penetrate the BBB non-invasively via passive transcellular diffusion when administered orally or intravenously.

However, brain exposure is not necessarily uniform or sustained and is affected by systemic clearance, plasma protein binding, and active efflux by transporters such as P-glycoprotein (P-gp). Depending on compound pharmacokinetics and target engagement, repeated dosing may be required [44,138], with pharmacodynamic effects depending on target engagement, tissue exposure, and compound persistence in the brain.

This difference in delivery route between ASOs and SMCs is reflected in a corresponding difference in intracellular half-life.

Finally, ASOs achieve high sequence specificity through Watson–Crick base pairing, which limits sequence-dependent off-target effects but does not eliminate hybridisation-independent toxicities, including innate immune activation via Toll-like receptors, non-specific protein binding, and neuroinflammation, which are not predictable from sequence complementarity alone [120].

In contrast, SMCs depend on structure-based recognition and may interact with unintended RNA structures or proteins sharing motifs similar to the MAPT exon 10–intron 10 stem-loop, potentially leading to broader alterations in splicing networks and therefore requiring thorough evaluation of target engagement and off-target effects [44,116,138]. Table 3 summarises the main pharmacological differences between the two approaches.

## 6. Translation of MAPT RNA Therapies

Recent advances in tau-targeted RNA therapeutics are driving the translation of MAPT exon 10 splicing therapies from preclinical development into human studies. Approaches targeting exon 10, including ENA-ASOs and small-molecule splicing modulators (SMCs), remain in preclinical or early clinical stages for FTDP-17.

However, studies involving MAPT RNA therapies in humans have set a significant precedent for translation. Diranersen (BIIB080), an RNase H1-active gapmer ASO targeting MAPT mRNA in individuals with early Alzheimer’s disease, has been shown to reduce CSF total tau levels in a dose-dependent manner and to be safe and well tolerated in earlier clinical studies [88]. Other MAPT RNA therapies are also advancing in clinical trials, including the siRNA LY3954068, which is currently in Phase 1 for the treatment of early-stage Alzheimer’s disease and is administered intrathecally, and the ASO NIO752, which has progressed to Phase 3 evaluation in the treatment of progressive supranuclear palsy [140,141].

Further translational evidence comes from the 18-month randomised placebo-controlled Phase 2 CELIA trial (NCT05399888) of Diranersen, involving 416 participants with mild cognitive impairment or mild Alzheimer’s disease dementia [142]. 

Although the trial did not show a linear dose–response relationship for CDR-SB, all tested treatments substantially reduced CSF total tau and Tau-PET signal. The lowest dose (60 mg every six months) showed the most consistent clinical benefit across several cognitive measures [142,143].

For FTDP-17, clinical translation requires a more precise framework because the disease is driven by pathogenic MAPT mutations that directly alter exon 10 splicing. 

Future trials should incorporate genetic pre-screening and stratification by the underlying molecular mechanism, distinguishing intronic mutations that destabilise the exon 10–intron 10 stem-loop (e.g., IVS10+16 and IVS10+14) from exonic variants that alter splicing regulatory elements (e.g., N279K and S305N).

This difference could affect therapeutic design, target accessibility and dosage requirements of steric-blocking ASOs or structure-directed SMCs. Early treatments can also be particularly relevant for genetically defined FTDP-17, where treatment could potentially begin before substantial tau pathology and neuronal loss occur. A useful precedent is provided by nusinersen, a splice-switching ASO used to treat spinal muscular atrophy. Presymptomatic treatment of infants with this condition who are genetically diagnosed has been shown to result in markedly improved developmental and motor outcomes, demonstrating the potential value of intervening before clinical disease onset in inherited splicing disorders [144].

While these clinical experiences do not yet demonstrate the efficacy of an exon 10-specific therapy in FTDP-17, they demonstrate the clinical feasibility of CNS-directed RNA therapeutics. They also provide frameworks for future pharmacological and biomarker studies and support a precision medicine approach that combines mutation-specific splicing correction with appropriate CNS delivery, early intervention, and longitudinal molecular monitoring.

## 7. CNS Delivery Challenges and Future Perspectives

**Figure 3:**
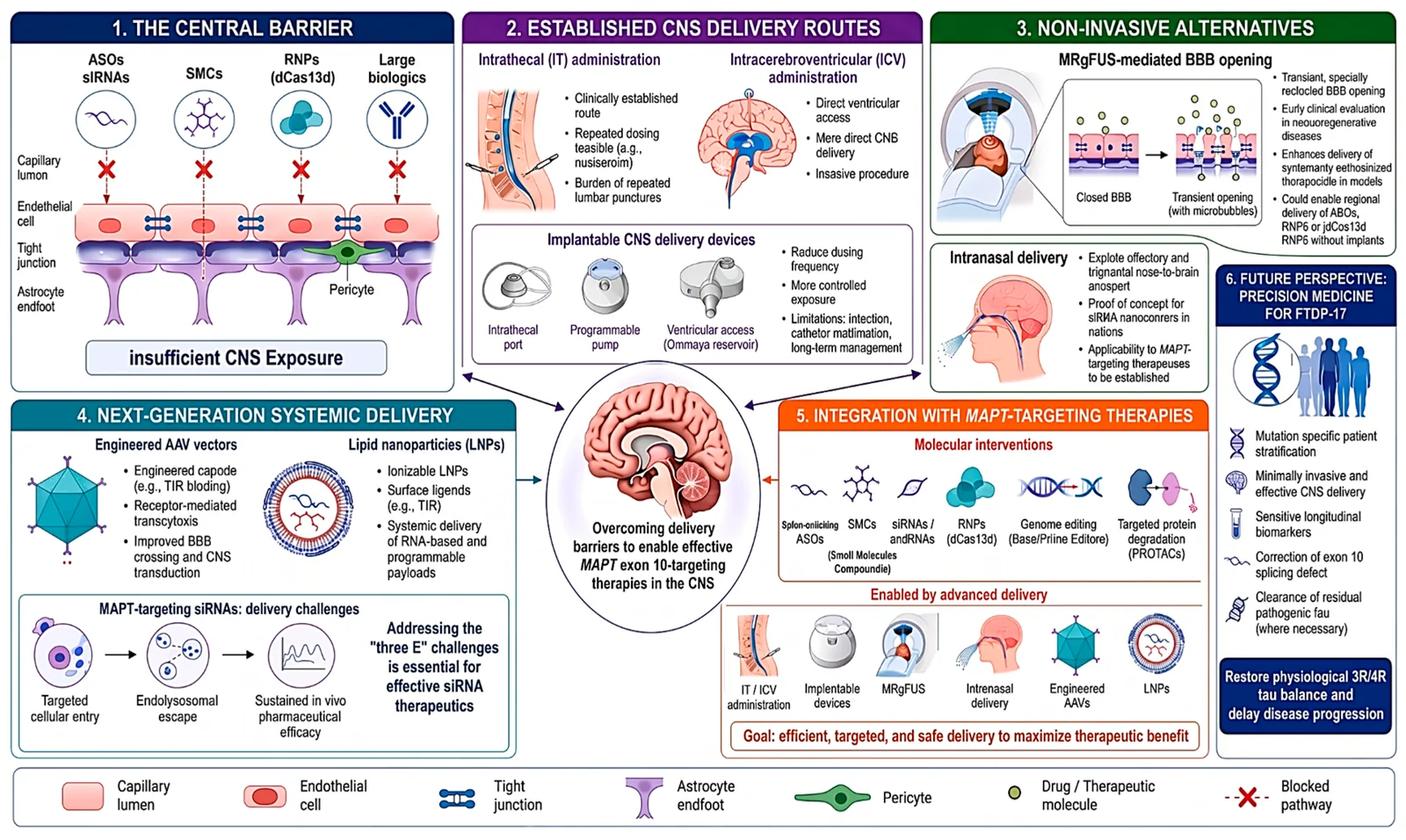
Challenges and new strategies for delivering *MAPT* exon 10 therapies to the CNS in FTDP-17. (1) The blood–brain barrier (BBB) blocks the passive entry of ASOs, siRNAs, and SMCs into the CNS. (2) Intrathecal and intracerebroventricular administration, as well as devices like ports, pumps, and Ommaya reservoirs, provide direct CNS access but are invasive. (3) Non-invasive systems such as MRgFUS and intranasal delivery offer alternative routes for CNS delivery. (4) Next-generation systemic platforms, including AAV vectors and receptor-targeted LNPs, improve BBB crossing and address the “three E” delivery barriers. (5) Advanced delivery technologies combined with therapies that correct *MAPT* exon 10 alternative splicing. (6) This approach supports future precision medicine for FTDP-17 by restoring the 3R/4R tau ratio.

Effective treatment of FTDP-17 requires sustained delivery of molecular therapies to the central nervous system (CNS), which makes the blood–brain barrier (BBB) a major obstacle to the clinical translation of approaches targeting MAPT exon 10 [86,136,139]. CNS delivery is a particular bottleneck for hydrophilic nucleic acid therapeutics. Molecules targeting MAPT, such as ASOs and siRNAs, exhibit limited passive permeability across the BBB. Systemic administration generally results in insufficient CNS uptake (Figure 3, Panel 1). Therefore, intrathecal (IT) administration remains the main clinically established method, with intracerebroventricular (ICV) administration as a more direct alternative [85,145,146].

The clinical use of IT splice-switching ASOs, exemplified by nusinersen for spinal muscular atrophy, demonstrates that repeated CNS delivery of ASOs is feasible [87], although repeated administration represents a substantial burden for patients requiring life-long treatment (Figure 3, Panel 2).

Implantable systems, intrathecal ports, programmable pumps, and ventricular devices (e.g., the Ommaya reservoir) are being investigated to reduce dosing frequency and control CNS exposure. However, infection, catheter malfunction, and long-term device management remain important limitations [145,146,147] (Figure 3, Panel 2).

Non-invasive alternatives are also being evaluated for FTDP-17. Among these, one possibility is intranasal administration, which exploits direct olfactory and trigeminal nose-to-brain transport and has been demonstrated to be a non-invasive route for siRNA nanocarriers in proof-of-concept rodent studies [148] (Figure 3, Panel 3).

Another option is magnetic resonance-guided focused ultrasound (MRgFUS) combined with microbubbles, which can temporarily open the BBB in a specific brain area (Figure 3, Panel 3). This technique has been tested in early clinical trials for neurodegenerative diseases [149] and can improve the delivery of tau-directed therapeutics administered via the bloodstream to the brain [150].

Although not yet applied specifically to MAPT exon 10-targeting therapies, MRgFUS could facilitate local delivery of ASOs, SMCs, or dCas13d ribonucleoprotein complexes. This system would remove the need for permanent implants.

After the initial demonstration that direct intracerebral administration can reduce pathological tau, subsequent approaches explored chemically stabilised, ligand-conjugated siRNAs and AAV-delivered artificial microRNAs (amiRNAs) to enhance persistence and CNS exposure (Figure 3, Panel 4). However, these platforms still need to overcome the broader ‘three E’ challenges recently proposed for siRNA drug development: targeted cellular entry, endolysosomal escape, and sustained *in vivo* pharmaceutical efficacy [101]. Importantly, AAV capsids engineered to bind specific endothelial receptors (e.g., the transferrin receptor) and facilitate receptor-mediated transcytosis, as well as lipid nanoparticles (LNPs) functionalised to do the same, are being developed to enable crossing of the BBB. These two approaches could facilitate the less-invasive delivery of RNA-based, programmable therapeutic platforms relevant to FTDP-17 [130].

Future MAPT exon 10 therapies will need to combine advances in delivery with precise molecular treatments. Using AI and machine learning in drug design, along with transcriptomic screening, could make SMCs more selective by reducing unwanted interactions with other splicing factors [116,137]. Targeted delivery of programmable RNA tools, such as CRISPR-dCas13d, may enable temporary changes in splicing without altering the genome permanently [24,130]. Targeted protein degradation offers another treatment option. For example, PROTACs can selectively remove abnormal tau from neurons taken from FTD patients [151].

Instead of offering separate treatments, these approaches highlight the importance of combining molecular correction with effective delivery to the central nervous system and, when needed, removing tau (Figure 3, Panel 5). This focus on splicing is part of a growing group of tau-targeted therapies, which also includes immunotherapy, aggregation inhibitors, and microtubule stabilisers, as reviewed elsewhere [152].

These developments help build a precision-medicine approach for FTDP-17. This approach combines exon 10 correction, efficient, less invasive CNS delivery, and removal of residual tau (Figure 3, Panel 6). It aims to restore the normal 3R/4R ratio and potentially help slow disease progression.

## 8. Conclusions

Over the past thirty years, research has identified the genetic and post-transcriptional mechanisms behind the MAPT alternative splicing link to FTDP-17. They showed that regulating exon 10 alternative splicing could effectively treat tau-related diseases. Switching from older models, such as minigenes and cell lines (e.g., SH-SY5Y, PC12, and NSC-34), to more realistic systems has been crucial. Moreover, patient-derived 2D neurons, 3D brain organoids, and engineered hydrogels fill the gap left by rodent models, which lack human splicing patterns.

Combining humanised mouse models with 3D human organoids enables researchers to observe how a splicing error leads to toxic tau protein accumulation in living tissue. These new models have sped up the development of targeted RNA and DNA therapies. The focus is shifting from treating symptoms to altering the disease course with precision medicine.

Preclinical testing shows splice-switching ASOs can restore tau balance in living systems. Oral SMCs offer a non-invasive option that crosses the blood–brain barrier. SMaRT and RNA stem-loop binders offer other ways to correct splicing after transcription. New CRISPR-based tools, including reversible transcript editing (dCas13d) and permanent genome editing, treat the disease at RNA and DNA levels. Recent progress, such as the Phase 2 CELIA trial of Diranersen, demonstrates the potential of Tau-PET imaging and plasma biomarkers (p-Tau217/231) as surrogate endpoints. These data could speed approval of targeted drugs for FTDP-17 [142,143,153].

However, several challenges remain before these molecular therapies can be used in clinical stages. First, delivery systems need improvement, including enhancing dual-vector AAV systems, designing non-viral lipid nanoparticles, and reducing off-target effects.

Developing implantable devices for patients is also necessary [130,146,147]. Second, long-term biomarkers, such as digital Tau-PET imaging and sensitive biofluid tests, are required to identify patients and track early treatment response [153]. Third, clinical trials should include accurate genetic screening of patients, since different FTDP-17 variants affect MAPT exon 10 splicing through distinct molecular mechanisms.

In summary, combining basic RNA biology with advanced human disease models and novel delivery methods offers a promising approach to correcting MAPT exon 10 alternative splicing. This systemic approach could eventually lead to useful treatments for FTDP-17 and other tau-related diseases, significantly improving patients’ lives.

## Figures and Tables

**Table 1:** Intronic and exonic *MAPT* gene mutations affecting exon 10 alternative splicing in FTDP-17, including both isoform directions. Mutations discussed in the main text are shown in **bold**; the remaining variants are listed for completeness.

Mutation	Location	Type	Effect on Exon 10 Splicing	Variant Class	Potentially Compatible Strategy	Key Reference(s)
E9+33 G>C	Intron 9	Intronic	Alters exon 10 splicing (direction not fully characterised)	Transversion	Prime Editing	[14]
***IVS9-10 G>T***	Intron 9 (5′ of exon 10)	Intronic	↑ Exon 10 inclusion	Transversion	Prime Editing	[28]
*L266V*	Exon 9 (missense)	Exonic (distal)	↓ Exon 10 inclusion	Transversion	Prime Editing	[14]
*G272V*	Exon 9 (missense)	Exonic (distal)	↓ Exon 10 inclusion	Transversion	Prime Editing	[14]
***IVS10+3 G>A***	Intron 10 (ISS)	Intronic	↑ Exon 10 inclusion (stem-loop destabilisation)	Transition	ABE	[19]
***IVS10+11 T>C***	Intron 10 (ISS)	Intronic	↑ Exon 10 inclusion (stem-loop destabilisation)	Transition	CBE	[19]
IVS10+12 C>T	Intron 10 (ISS)	Intronic	↑ Exon 10 inclusion (stem-loop destabilisation)	Transition	ABE	[14]
***IVS10+13 A>G***	Intron 10 (ISS)	Intronic	↑ Exon 10 inclusion (stem-loop destabilisation)	Transition	CBE	[3,20]
***IVS10+14 C>T***	Intron 10 (ISS)	Intronic	↑ Exon 10 inclusion (stem-loop destabilisation)	Transition	ABE	[3,19,29]
***IVS10+16 C>T***	Intron 10 (ISS)	Intronic	↑ Exon 10 inclusion (stem-loop destabilisation); most common FTDP-17 mutation	Transition	ABE	[3,19,29]
IVS10+19 C>G	Intron 10 (ISM)	Intronic	↓ Exon 10 inclusion (opposite effect to ISS mutations)	Transversion	Prime Editing	[14]
IVS10+29 G>A	Intron 10	Intronic	↓ Exon 10 inclusion	Transition	ABE	[14]
***N279K***	Exon 10 (missense, PPE)	Exonic	↑ Exon 10 inclusion (strengthens ESE, recruits Tra2β)	Transversion	Prime Editing	[21,30]
*N296H*	Exon 10 (missense, ESS)	Exonic	↑ Exon 10 inclusion	Transversion	Prime Editing	[31,32]
*N296N*	Exon 10 (silent, ESS)	Exonic	↑ Exon 10 inclusion	Transition	CBE	[31]
*ΔN296*	Exon 10 (deletion, ESS)	Exonic	↑ Exon 10 inclusion	Small deletion	Prime Editing	[14]
***L284L***	Exon 10 (silent, ACE)	Exonic	↑ Exon 10 inclusion	Transition	CBE	[31]
*P301S*	Exon 10 (missense, ESE)	Exonic	↑ Exon 10 inclusion	Transition	ABE	[14]
*G303V*	Exon 10 (missense, ESE)	Exonic	↑ Exon 10 inclusion	Transversion	Prime Editing	[14]
***S305N***	Exon 10 (missense)	Exonic	↑ Exon 10 inclusion (destabilises stem-loop)	Transition	ABE	[31]
***S305I***	Exon 10 (missense)	Exonic	↑ Exon 10 inclusion	Transversion	Prime Editing	[31]
***S305S***	Exon 10 (silent)	Exonic	↑ Exon 10 inclusion (weakens 5′ splice site)	Transition	CBE	[31]
***ΔK280***	Exon 10 (deletion, PPE)	Exonic	↓ Exon 10 inclusion (disrupts purine-rich ESE; opposite effect)	Small deletion	Prime Editing	[31,33]

Note: P301L, a well-known exon 10 missense mutation, is deliberately excluded from this table because it is a gain-of-function mutation affecting tau protein aggregation propensity rather than exon 10 splicing itself [34]. C>T/G>A transitions are assigned to ABE, T>C/A>G to CBE, and transversions or small deletions to Prime Editing. These assignments are theoretical and do not imply experimentally validated correction, as actual editability depends on PAM availability, strand orientation, editing window, bystander editing, and locus-specific activity.

**Table 2:** Comparative therapeutic approaches for MAPT exon 10 mis-splicing.

Therapeutic Approach	Mechanism of Action	Validated Experimental Models	Main Advantages	Current Challenges/Limitations	Key References
Antisense Oligonucleotides (ASOs)	Bind MAPT pre-mRNA at exon 10 splicing sites to block inclusion or promote targeted exon skipping sterically	hiPSC-derived neurons; hTau and humanised transgenic mice; clinical-stage data from Diranersen/MAPTRx (Section 6)	High sequence specificity; clinically validated drug platform (e.g., Nusinersen)	Requires periodic invasive intrathecal administration; gradient-dependent brain distribution	[83,86,87,88,103,104,107]
RNA Interference (siRNA/amiRNA)	Double-stranded RNA loaded into RISC guides catalytic, sequence-specific cleavage of MAPT mRNA, lowering total tau	P301S transgenic mice (intrahippocampal delivery); AAV-amiRNA preclinical models	Catalytic, multi-turnover knockdown; durable effect with vector-delivered amiRNA; clinical platform validated by 6 FDA-approved siRNA drugs (non-CNS indications)	Lowers total tau rather than correcting the 3R/4R splice ratio; delivery/BBB constraints similar to ASOs	[89,91,93,95,96,97,98]
Small Molecule Splicing Modulators (SMCs)	Orally bioavailable compounds that bind the pre-mRNA stem-loop or modulate SR-protein/RNP affinity	Minigene screening; hiPSC neurons; gene-replacement (N279K) and hTau knock-in mouse models	Brain-penetrant (crosses BBB); oral administration; high patient compliance	Off-target risk is structural rather than sequence-based; potential long-term toxicity not yet fully characterised; potential long-term toxicity not yet fully characterised	[44,45,109,110,111,112,113,114,115,116]
Spliceosome-Mediated RNA *Trans*-Splicing (SMaRT)	Replaces the mutated exon 10 tract at the pre-mRNA level with an engineered exogenous PTM sequence	HEK293 cell lines; primary neuronal cultures; symptomatic transgenic mouse models	Highly specific correction at the post-transcriptional level; native promoter control preserved	Low *trans*-splicing efficiency *in vivo*; challenges related to AAV/lentiviral vector delivery and immunogenicity	[120,121,122,123,124]
CRISPR-dCas13d Transcript Engineering	Catalytically dead Cas13d + gRNA sterically blocks intronic splicing enhancers (e.g., MBNL sites)	*In vitro* transcript-engineering studies	Programmable; reversible; no permanent DNA modification	Efficacy currently demonstrated mainly *in vitro*; delivery to CNS is still to be optimised	[24]
CRISPR Base/Prime Editing	Direct editing of genome using base editors (CBE/ABE) for compatible transition mutations, or prime editing for transversions and small deletions, depending on the specific nucleotide change (Table 1)	Human iPSC lines (precedent in other Mendelian disorders); *in vivo* prime/base editing demonstrated in post-mitotic neurons for other neurodegenerative disorders (not yet applied specifically to MAPT)	Provides a permanent, one-time cure by correcting the genetic defect at its root	Difficult delivery into the post-mitotic adult brain; risk of stable off-target genomic modifications; editing strategy is mutation-dependent (base editing requires a compatible transition, PAM, and editing window); no MAPT-specific study yet published	[84,85,126,127,128,129]
RNA Stem-Loop Binders	Aptamers/aminoglycoside derivatives stabilise the loosened intron 10 Stem-Loop, restoring 5′ splice-site occlusion.	Biochemical and structural RNA-binding studies	Structure-first approach; potential small-molecule druggability	Early-stage preclinical development; specificity for the mutant vs wild-type stem-loop still to be optimised	[109,131]

Note: Compatibility of base editing (CBE/ABE) versus prime editing for individual FTDP-17 variants, based on nucleotide change (transition, transversion, or deletion), is detailed in Table 1. Abbreviations: AAV, adeno-associated virus; ASOs, antisense oligonucleotides; BBB, blood–brain barrier; CNS, central nervous system; gRNA, guide RNA; hiPSC, human induced pluripotent stem cell; MAPT, microtubule-associated protein tau; MBNL, muscleblind-like; PTM, pre-*trans*-splicing molecule; RNP, ribonucleoprotein; SMCs, small-molecule splicing modulators.

**Table 3:** Preclinical pharmacology of ENA/MOE antisense oligonucleotides (ASOs) versus small-molecule splicing modulators (SMCs).

Pharmacological Parameter	ENA/MOE Antisense Oligonucleotides (ASOs)	Small-Molecule Modulators (SMCs)	Key References
Molecular mass/nature	Large (~7–10 kDa), hydrophilic oligonucleotide	Small (~300–500 Da), lipophilic compound	[44,105]
BBB permeability	Impermeable (requires direct CNS injection)	Highly permeable (passive diffusion)	[44,139]
Primary route	Intrathecal (IT)/intracerebroventricular (ICV)	Oral (*per os*)/intravenous (IV)	[44,105]
Brain tissue distribution	Gradient-dependent; high in cortex/hippocampus; higher exposure in regions accessible from CSF, with variable penetration into deeper structures	Potentially broad; cortex and deep nuclei; regional exposure depends on pharmacokinetics, tissue distribution and efflux	[44,105]
Mechanism of action	Sequence-specific Watson–Crick steric blocking	Structure-specific binding to RNA/RNP complexes	[44,105]
Target affinity basis	Linear nucleotide base-pairing complementarity	Spatial pocket binding and conformational fit	[44,111]
Intracellular stability	Extremely high (months; nuclease-resistant)	Low to moderate (hours to days; metabolised)	[44,103,105]
Dosing regimen	Intermittent bolus (e.g., every 12–24 weeks)	Continuous, chronic (daily administration)	[44,103]
Off-target liability	Sequence-dependent hybridization off-targets; additional hybridization-independent toxicities, including immune activation and non-specific protein interactions	Theoretical structure-based off-target risk; optimised series show high specificity in the reported transcriptome-wide analysis (<1%)	[44,116,138]

## Data Availability

No new clinical or primary experimental biological data were created or analysed in this study. Data sharing is not applicable to this article.

## Abbreviations

The following abbreviations are used in this manuscript:

**Abbreviation**	**Full Term**
3R/4R	3-Repeat/4-Repeat Tau protein isoforms
ASO	Antisense Oligonucleotide
BBB	Blood–Brain Barrier
CNS	Central Nervous System
CSF	Cerebrospinal Fluid
ENA	2′-*O*,4′-*C*-ethylene-bridged nucleic acid
ESE/ESS	Exonic Splicing Enhancer/Silencer
FTDP-17	Frontotemporal Dementia and Parkinsonism linked to chromosome 17
FUS	Fused in Sarcoma
hiPSC	Human Induced Pluripotent Stem Cell
HTS	High-Throughput Screening
IT	Intrathecal
MAPT	Microtubule-Associated Protein Tau
MBNL	Muscleblind-Like Splicing Regulator
MOE	2′-*O*-methoxyethyl
MTBRs	Microtuble Binding Repeats
PET	Positron Emission Tomography
p-Tau	Hyperphosphorylated Tau protein
PTM	Pre-*Trans*-Splicing RNA Molecule
SFPQ	Splicing Factor Proline- and Glutamine-Rich
SMC	Small-Molecule Splicing Modulator
SMaRT	Spliceosome-Mediated RNA *Trans*-Splicing

## References

[1] Goedert M., Spillantini M.G., Jakes R., Rutherford D., Crowther R.A. (1989). Multiple isoforms of human microtubule-associated protein tau: Sequences and localization in neurofibrillary tangles of Alzheimer’s disease. Neuron.

[2] Goedert M., Spillantini M.G. (2006). A century of Alzheimer’s disease and tau pathology. Science.

[3] Hutton M., Lendon C.L., Rizzu P., Baker M., Froelich S., Houlden H., Pickering-Brown S., Chakraverty S., Isaacs A., Grover A. (1998). Association of missense and 5’-splice-site mutations in tau with the inherited dementia FTDP-17. Nature.

[4] Spillantini M.G., Murrell J.R., Goedert M., Farlow M.R., Klug A., Ghetti B. (1998). Mutation in the tau gene in familial multiple system tauopathy with presenile dementia. Proc. Natl. Acad. Sci. USA.

[5] Buée L., Delacourte A. (1999). Comparative biochemistry of tau in progressive supranuclear palsy, corticobasal degeneration, FTDP-17 and Pick’s disease. Brain Pathol..

[6] Arai T., Ikeda K., Akiyama H., Shikamoto Y., Tsuchiya K., Yagishita S., Beach T., Rogers J., Schwab C., McGeer P.L. (2001). Distinct isoforms of tau aggregated in neurons and glial cells in brains of patients with Pick’s disease, corticobasal degeneration and progressive supranuclear palsy. Acta Neuropathol..

[7] Bowles K.R., Pugh D.A., Oja L.M., Jadow B.M., Farrell K., Whitney K., Sharma A., Cherry J.D., Raj T., Pereira A.C. (2022). Dysregulated coordination of *MAPT* exon 2 and exon 10 splicing underlies different tau pathologies in PSP and AD. Acta Neuropathol..

[8] Neve R.L., Harris P., Kosik K.S., Kurnit D.M., Donlon T.A. (1986). Identification of cDNA clones for the human microtubule-associated protein tau and chromosomal localization of the genes for tau and microtubule-associated protein 2. Brain Res..

[9] Parra Bravo C., Naguib S.A., Gan L. (2024). Cellular and pathological functions of tau. Nat. Rev. Mol. Cell Biol..

[10] Andreadis A., Brown W.M., Kosik K.S. (1992). Structure and novel exons of the human tau gene. Biochemistry.

[11] Andreadis A. (2005). Tau gene alternative splicing: Expression patterns, regulation and modulation of function in development and disease. Biochim. Biophys. Acta (BBA) Mol. Basis Dis..

[12] Buée L., Bussière T., Buée-Scherrer V., Delacourte A., Hof P.R. (2000). Tau protein isoforms, phosphorylation and role in neurodegenerative disorders. Brain Res. Rev..

[13] Wang Y., Mandelkow E. (2016). Tau in physiology and pathology. Nat. Rev. Neurosci..

[14] Liu F., Gong C.X. (2008). Tau exon 10 alternative splicing and tauopathies. Mol. Neurodegener..

[15] Goode B.L., Chau M., Denis P.E., Feinstein S.C. (2000). Structural and functional differences between 3-repeat and 4-repeat tau isoforms. J. Biol. Chem..

[16] Kosik K.S., Orecchio L.D., Bakalis S., Neve R.L. (1989). Developmentally regulated expression of specific tau sequences. Neuron.

[17] García-Escudero V., Ruiz-Gabarre D., Gargini R., Pérez M., García E., Cuadros R., Hernández I.H., Cabrera J.R., García-Escudero R., Lucas J.J. (2021). A new non-aggregative splicing isoform of human Tau is decreased in Alzheimer’s disease. Acta Neuropathol..

[18] D’Souza I., Schellenberg G.D. (2000). Determinants of 4-repeat tau expression. Coordination between enhancing and inhibitory splicing sequences for exon 10 inclusion. J. Biol. Chem..

[19] Grover A., Houlden H., Baker M., Adamson J., Lewis J., Prihar G., Pickering-Brown S., Duff K., Hutton M. (1999). 5’ splice site mutations in tau associated with the inherited dementia FTDP-17 affect a stem-loop structure that regulates alternative splicing of exon 10. J. Biol. Chem..

[20] Pickering-Brown S.M., Richardson A.M.T., Snowden J.S., McDonagh A.M., Burns A., Braude W., Baker M., Liu W.K., Yen S.H., Hardy J. (2002). Inherited frontotemporal dementia in nine British families associated with intronic mutations in the tau gene. Brain.

[21] Jiang Z., Tang H., Havlioglu N., Zhang X., Stamm S., Yan R., Wu J.Y. (2003). Mutations in tau gene exon 10 associated with FTDP-17 alter the activity of an exonic splicing enhancer to interact with Tra2 beta. J. Biol. Chem..

[22] Glatz D.C., Rujescu D., Tang Y., Berendt F.J., Hartmann A.M., Faltraco F., Rosenberg C., Hulette C., Jellinger K., Hampel H. (2006). The alternative splicing of tau exon 10 and its regulatory proteins CLK2 and TRA2-BETA1 changes in sporadic Alzheimer’s disease. J. Neurochem..

[23] Aranda-Abreu G.E., Rojas-Durán F., Hernández-Aguilar M.E., Herrera-Covarrubias D., Tlapa-Monge L.R., Mestizo-Gutiérrez S.L. (2026). Alternative splicing in Alzheimer’s disease: Driver, modifier, or consequence of neurodegeneration. Mech. Ageing Dev..

[24] Recinos Y., Bao S., Wang X., Phillips B.L., Yeh Y.-T., Weyn-Vanhentenryck S.M., Swanson M.S., Zhang C. (2024). Lineage-specific splicing regulation of *MAPT* gene in the primate brain. Cell Genom..

[25] Andorfer C., Kress Y., Espinoza M., de Silva R., Tucker K.L., Barde Y.A., Duff K., Davies P. (2003). Hyperphosphorylation and aggregation of tau in mice expressing normal human tau isoforms (htau mice). J. Neurochem..

[26] Bak A., Koch H., van Loo K.M., Schmied K., Gittel B., Weber Y., Ort J., Schwarz N., Tauber S.C., Wuttke T.V. (2024). Human organotypic brain slice cultures: A detailed and improved protocol for preparation and long-term maintenance. J. Neurosci. Methods.

[27] Joseph K., Vasilikos I., Grauvogel J., Shah M.J., Reinacher P.C., Nakagawa J.M., Häussler U., Straehle J., Neidert N.N., Fistouris P. (2025). Human brain slice cultures: Translational applications and ethical considerations. Life Sci. Alliance.

[28] Olszewska D.A., Fearon C., McGuigan C., McVeigh T.P., Houlden H., Polke J.M., Lawlor B., Coen R., Hutchinson M., Hutton M. (2021). A clinical, molecular genetics and pathological study of a FTDP-17 family with a heterozygous splicing variant c.823-10G\>T at the intron 9/exon 10 of the MAPT gene. Neurobiol. Aging.

[29] Jiang Z., Cote J., Kwon J.M., Goate A.M., Wu J.Y. (2000). Aberrant splicing of tau pre-mRNA caused by intronic mutations associated with the inherited dementia frontotemporal dementia with parkinsonism linked to chromosome 17. Mol. Cell. Biol..

[30] Hasegawa M., Smith M.J., Iijima M., Tabira T., Goedert M. (1999). FTDP-17 mutations N279K and S305N in tau produce increased splicing of exon 10. FEBS Lett..

[31] D’Souza I., Poorkaj P., Hong M., Nochlin D., Lee V.M., Bird T.D., Schellenberg G.D. (1999). Missense and silent tau gene mutations cause frontotemporal dementia with parkinsonism-chromosome 17 type, by affecting multiple alternative RNA splicing regulatory elements. Proc. Natl. Acad. Sci. USA.

[32] Iseki E., Matsumura T., Marui W., Hino H., Odawara T., Sugiyama N., Suzuki K., Sawada H., Arai T., Kosaka K. (2001). Familial frontotemporal dementia and parkinsonism with a novel N296H mutation in exon 10 of the tau gene and a widespread tau accumulation in the glial cells. Acta Neuropathol..

[33] Verheyen A., Diels A., Reumers J., Van Hoorde K., Van den Wyngaert I., van Outryve d’Ydewalle C., De Bondt A., Kuijlaars J., De Muynck L., De Hoogt R. (2018). Genetically engineered iPSC-derived FTDP-17 MAPT neurons display mutation-specific neurodegenerative and neurodevelopmental phenotypes. Stem Cell Rep..

[34] Hong M., Zhukareva V., Vogelsberg-Ragaglia V., Wszolek Z., Reed L., Miller B.I., Geschwind D.H., Bird T.D., McKeel D., Goate A. (1998). Mutation-specific functional impairments in distinct tau isoforms of hereditary FTDP-17. Science.

[35] Tsuboi Y., Baker M., Hutton M.L., Uitti R.J., Rascol O., Delisle M.B., Soulages X., Murrell J.R., Ghetti B., Yasuda M. (2002). Clinical and genetic studies of families with the tau N279K mutation (FTDP-17). Neurology.

[36] Tsuboi Y., Uitti R.J., Delisle M.B., Ferreira J.J., Brefel-Courbon C., Rascol O., Ghetti B., Murrell J.R., Hutton M., Baker M. (2002). Clinical features and disease haplotypes of individuals with the N279K tau gene mutation: A comparison of the pallidopontonigral degeneration kindred and a French family. Arch. Neurol..

[37] Kampers T., Friedhoff P., Biernat J., Mandelkow E.M., Mandelkow E. (1996). RNA stimulates aggregation of microtubule-associated protein tau into Alzheimer-like paired helical filaments. FEBS Lett..

[38] Wang J., Gao Q.S., Wang Y., Lafyatis R., Stamm S., Andreadis A. (2004). Tau exon 10, whose missplicing causes frontotemporal dementia, is regulated by an intricate interplay of cis elements and trans factors. J. Neurochem..

[39] Corsi A., Bombieri C., Valenti M.T., Romanelli M.G. (2022). Tau isoforms: Gaining insight into *MAPT* alternative splicing. Int. J. Mol. Sci..

[40] Cooper T.A. (2005). Use of minigene systems to dissect alternative splicing elements. Methods.

[41] Stoss O., Stoilov P., Hartmann A.M., Nayler O., Stamm S. (1999). The in vivo minigene approach to analyze tissue-specific splicing. Brain Res. Protoc..

[42] Desviat L.R., Pérez B., Ugarte M. (2012). Minigenes to confirm exon skipping mutations. Exon Skipping: Methods and Protocols.

[43] Yu Q., Guo J., Zhou J. (2004). A minimal length between tau exon 10 and 11 is required for correct splicing of exon 10. J. Neurochem..

[44] Silva M.C., Lindmeier H., Pigini P., Laughlin J., Yu Y., Morrill C., Arnold M.A., Barraza S.J., Saadipour K., Dieterich M. (2026). MAPT splicing modulators reduce 4R tau and rescue tauopathy phenotypes in human neurons and in a mouse model. Sci. Transl. Med..

[45] Stoilov P., Lin C.H., Damoiseaux R., Nikolic J., Black D.L. (2008). A high-throughput screening strategy identifies cardiotonic steroids as alternative splicing modulators. Proc. Natl. Acad. Sci. USA.

[46] Covello G., Siva K., Adami V., Denti M.A. (2023). HCS-Splice: A high-content screening method to advance the discovery of RNA splicing-modulating therapeutics. Cells.

[47] Xicoy H., Wieringa B., Martens G.J.M. (2017). The SH-SY5Y cell line in Parkinson’s disease research: A systematic review. Mol. Neurodegener..

[48] Shipley M.M., Mangold C.A., Szpara M.L. (2016). Differentiation of the SH-SY5Y human neuroblastoma cell line. J. Vis. Exp..

[49] Agholme L., Lindström T., Kågedal K., Marcusson J., Hallbeck M. (2010). An in vitro model for neuroscience: Differentiation of SH-SY5Y cells into cells with morphological and biochemical characteristics of mature neurons. J. Alzheimers Dis..

[50] Dravid A., Raos B., Svirskis D., O’Carroll S.J. (2021). Optimised techniques for high-throughput screening of differentiated SH-SY5Y cells and application for neurite outgrowth assays. Sci. Rep..

[51] Greene L.A., Tischler A.S. (1976). Establishment of a noradrenergic clonal line of rat adrenal pheochromocytoma cells which respond to nerve growth factor. Proc. Natl. Acad. Sci. USA.

[52] Kalbfuss B., Mabon S.A., Misteli T. (2001). Correction of alternative splicing of tau in frontotemporal dementia and parkinsonism linked to chromosome 17. J. Biol. Chem..

[53] Furukawa K., D’Souza I., Crudder C.H., Onodera H., Itoyama Y., Poorkaj P., Bird T.D., Schellenberg G.D. (2000). Pro-apoptotic effects of tau mutations in chromosome 17 frontotemporal dementia and parkinsonism. Neuroreport.

[54] Maier O., Böhm J., Dahm M., Brück S., Beyer C., Johann S. (2013). Differentiated NSC-34 motoneuron-like cells as experimental model for cholinergic neurodegeneration. Neurochem. Int..

[55] Ishigaki S., Fujioka Y., Okada Y., Okado H., Katsuno M., Sobue G. (2017). Altered tau isoform ratio caused by loss of FUS and SFPQ function leads to FTLD-like phenotypes. Cell Rep..

[56] Donato R., Miljan E.A., Hines S.J., Aouabdi S., Pollock K., Patel S., Edwards F.A., Sinden J.D. (2007). Differential development of neuronal physiological responsiveness in two human neural stem cell lines. BMC Neurosci..

[57] Sindi G., Ismael S., Uddin R., Slepchenko K.G., Colvin R.A., Lee D. (2024). Endogenous tau released from human ReNCell VM cultures by neuronal activity is phosphorylated at multiple sites. bioRxiv.

[58] Takahashi K., Tanabe K., Ohnuki M., Narita M., Ichisaka T., Tomoda K., Yamanaka S. (2007). Induction of pluripotent stem cells from adult human fibroblasts by defined factors. Cell.

[59] Bullmann T., de Silva R., Holzer M., Mori H., Arendt T. (2007). Expression of embryonic tau protein isoforms persist during adult neurogenesis in the hippocampus. Hippocampus.

[60] Hernández F., Merchán-Rubira J., Vallés-Saiz L., Rodríguez-Matellán A., Avila J. (2020). Differences between human and murine tau at the N-terminal end. Front. Aging Neurosci..

[61] Iovino M., Agathou S., González-Rueda A., Del Castillo Velasco-Herrera M., Borroni B., Alberici A., Lynch T., O’Dowd S., Geti I., Gaffney D. (2015). Early maturation and distinct tau pathology in induced pluripotent stem cell-derived neurons from patients with MAPT mutations. Brain.

[62] Imamura K., Sahara N., Kanaan N.M., Tsukita K., Kondo T., Kutoku Y., Ohsawa Y., Sunada Y., Kawakami K., Hotta A. (2016). Calcium dysregulation contributes to neurodegeneration in FTLD patient iPSC-derived neurons. Sci. Rep..

[63] Kopach O., Esteras N., Wray S., Abramov A.Y., Rusakov D.A. (2021). Genetically engineered *MAPT* 10+16 mutation causes pathophysiological excitability of human iPSC-derived neurons related to 4R tau-induced dementia. Cell Death Dis..

[64] Wren M.C., Zhao J., Liu C.C., Murray M.E., Atagi Y., Davis M.D., Fu Y., Okano H.J., Ogaki K., Strongosky A.J. (2015). Frontotemporal dementia-associated N279K tau mutant disrupts subcellular vesicle trafficking and induces cellular stress in iPSC-derived neural stem cells. Mol. Neurodegener..

[65] Ritter M.L., Avila J., García-Escudero V., Hernández F., Pérez M. (2018). Frontotemporal dementia-associated N279K tau mutation localizes at the nuclear compartment. Front. Cell. Neurosci..

[66] Sposito T., Preza E., Mahoney C.J., Setó-Salvia N., Ryan N.S., Morris H.R., Arber C., Devine M.J., Houlden H., Warner T.T. (2015). Developmental regulation of tau splicing is disrupted in stem cell-derived neurons from frontotemporal dementia patients with the 10+16 splice-site mutation in *MAPT*. Hum. Mol. Genet..

[67] Ehrlich M., Hallmann A.L., Reinhardt P., Araúzo-Bravo M.J., Korr S., Röpke A., Psathaki O.E., Ehling P., Meuth S.G., Oblak A.L. (2015). Distinct neurodegenerative changes in an induced pluripotent stem cell model of frontotemporal dementia linked to mutant TAU protein. Stem Cell Rep..

[68] Bowles K.R., Silva M.C., Whitney K., Bertucci T., Berlind J.E., Lai J.D., Garza J.C., Boles N.C., Mahali S., Strang K.H. (2021). ELAVL4, splicing, and glutamatergic dysfunction precede neuron loss in *MAPT* mutation cerebral organoids. Cell.

[69] Lancaster M.A., Renner M., Martin C.A., Wenzel D., Bicknell L.S., Hurles M.E., Homfray T., Penninger J.M., Jackson A.P., Knoblich J.A. (2013). Cerebral organoids model human brain development and microcephaly. Nature.

[70] Quadrato G., Nguyen T., Macosko E.Z., Sherwood J.L., Min Yang S., Berger D.R., Maria N., Scholvin J., Goldman M., Kinney J.P. (2017). Cell diversity and network dynamics in photosensitive human brain organoids. Nature.

[71] Miguel L., Rovelet-Lecrux A., Feyeux M., Frebourg T., Nassoy P., Campion D., Lecourtois M. (2019). Detection of all adult Tau isoforms in a 3D culture model of iPSC-derived neurons. Stem Cell Res..

[72] Cordella F., Mautone L., Salerno D., Tondo L., Ghirga S., D’Antoni C., Parente E., Romeo M.A., Cirone M., Bezzi P. (2025). Bezafibrate treatment rescues neurodevelopmental and neurodegenerative defects in 3D cortical organoid model of *MAPT* frontotemporal dementia. Alzheimers Dement..

[73] Nogueira G.O., Garcez P.P., Bardy C., Cunningham M.O., Sebollela A. (2022). Modeling the human brain with ex vivo slices and in vitro organoids for translational neuroscience. Front. Neurosci..

[74] Szebényi K., Wenger L.M.D., Sun Y., Dunn A.W.E., Limegrover C.A., Gibbons G.M., Conci E., Paulsen O., Mierau S.B., Balmus G. (2021). Human ALS/FTD brain organoid slice cultures display distinct early astrocyte and targetable neuronal pathology. Nat. Neurosci..

[75] Plug B.C., Revers I.M., Breur M., Muñoz González G., Timmerman J.A., Meijns N.R.C., Hamberg D., Wagendorp J., Nutma E., Wolf N.I. (2024). Human post-mortem organotypic brain slice cultures: A tool to study pathomechanisms and test therapies. Acta Neuropathol. Commun..

[76] DeVos S.L., Miller R.L., Schoch K.M., Holmes B.B., Kebodeaux C.S., Wegener A.J., Chen G., Shen T., Tran H., Nichols B. (2017). Tau reduction prevents neuronal loss and reverses pathological tau deposition and seeding in mice with tauopathy. Sci. Transl. Med..

[77] Umeda T., Yamashita T., Kimura T., Ohnishi K., Takuma H., Ozeki T., Takashima A., Tomiyama T., Mori H. (2013). Neurodegenerative disorder FTDP-17-related tau intron 10 +16C\>T mutation increases tau exon 10 splicing and causes tauopathy in transgenic mice. Am. J. Pathol..

[78] Lewis J., McGowan E., Rockwood J., Melrose H., Nacharaju P., Van Slegtenhorst M., Gwinn-Hardy K., Paul Murphy M., Baker M., Yu X. (2000). Neurofibrillary tangles, amyotrophy, and progressive motor disturbance in mice expressing a mutant (P301L) tau protein. Nat. Genet..

[79] Santacruz K., Lewis J., Spires T., Paulson J., Kotilinek L., Ingelsson M., Guimaraes A., DeTure M., Ramsden M., McGowan E. (2005). Tau suppression in a neurodegenerative mouse model improves memory function. Science.

[80] DeVos S.L., Goncharoff D.K., Chen G., Kebodeaux C.S., Yamada K., Stewart F.R., Schuler D.R., Maloney S.E., Wozniak D.F., Rigo F. (2013). Antisense reduction of tau in adult mice protects against seizures. J. Neurosci..

[81] Dawson H.N., Cantillana V., Chen L., Vitek M.P. (2007). The tau N279K exon 10 splicing mutation recapitulates frontotemporal dementia and parkinsonism linked to chromosome 17 tauopathy in a mouse model. J. Neurosci..

[82] Taniguchi T., Doe N., Matsuyama S., Kitamura Y., Mori H., Saito N., Tanaka C. (2005). Transgenic mice expressing mutant (N279K) human tau show mutation dependent cognitive deficits without neurofibrillary tangle formation. FEBS Lett..

[83] Wood M., Yin H., McClorey G. (2007). Modulating the expression of disease genes with RNA-based therapy. PLoS Genet..

[84] Gaudelli N.M., Komor A.C., Rees H.A., Packer M.S., Badran A.H., Bryson D.I., Liu D.R. (2017). Programmable base editing of A•T to G•C in genomic DNA without DNA cleavage. Nature.

[85] Anzalone A.V., Randolph P.B., Davis J.R., Sousa A.A., Koblan L.W., Levy J.M., Chen P.J., Wilson C., Newby G.A., Raguram A. (2019). Search-and-replace genome editing without double-strand breaks or donor DNA. Nature.

[86] Siva K., Covello G., Denti M.A. (2014). Exon-skipping antisense oligonucleotides to correct missplicing in neurogenetic diseases. Nucleic Acid Ther..

[87] Finkel R.S., Mercuri E., Darras B.T., Connolly A.M., Kuntz N.L., Kirschner J., Chiriboga C.A., Saito K., Servais L., Tizzano E. (2017). Nusinersen versus sham control in infantile-onset spinal muscular atrophy. N. Engl. J. Med..

[88] Mummery C.J., Börjesson-Hanson A., Blackburn D.J., Vijverberg E.G.B., De Deyn P.P., Ducharme S., Jonsson M., Schneider A., Rinne J.O., Ludolph A.C. (2023). Tau-targeting antisense oligonucleotide MAPTRx in mild Alzheimer’s disease: A phase 1b, randomized, placebo-controlled trial. Nat. Med..

[89] Fire A., Xu S., Montgomery M.K., Kostas S.A., Driver S.E., Mello C.C. (1998). Potent and specific genetic interference by double-stranded RNA in *Caenorhabditis elegans*. Nature.

[90] Hu B., Zhong L., Weng Y., Peng L., Huang Y., Zhao Y., Liang X.J. (2020). Therapeutic siRNA: State of the art. Signal Transduct. Target. Ther..

[91] Xu H., Rösler T.W., Carlsson T., de Andrade A., Fiala O., Höllerhage M., Oertel W.H., Goedert M., Aigner A., Höglinger G.U. (2014). Tau silencing by siRNA in the P301S mouse model of tauopathy. Curr. Gene Ther..

[92] Miller V.M., Xia H., Marrs G.L., Gouvion C.M., Lee G., Davidson B.L., Paulson H.L. (2003). Allele-specific silencing of dominant disease genes. Proc. Natl. Acad. Sci. USA.

[93] Miller V.M., Gouvion C.M., Davidson B.L., Paulson H.L. (2004). Targeting Alzheimer’s disease genes with RNA interference: An efficient strategy for silencing mutant alleles. Nucleic Acids Res..

[94] Covello G., Siva K., Denti M.A. (2016). RNA Interference Mediated Therapy for Neurodegenerative Diseases.

[95] Xiao B., Wang S., Pan Y., Zhi W., Gu C., Guo T., Zhai J., Li C., Chen Y.Q., Wang R. (2025). Development, opportunities, and challenges of siRNA nucleic acid drugs. Mol. Ther. Nucleic Acids.

[96] Adams D., Gonzalez-Duarte A., O’Riordan W.D., Yang C.C., Ueda M., Kristen A.V., Tournev I., Schmidt H.H., Coelho T., Berk J.L. (2018). Patisiran, an RNAi therapeutic, for hereditary transthyretin amyloidosis. N. Engl. J. Med..

[97] Scott L.J. (2020). Givosiran: First approval. Drugs.

[98] Scott L.J., Keam S.J. (2021). Lumasiran: First approval. Drugs.

[99] Santulli G., Jankauskas S.S., Gambardella J. (2021). Inclisiran: A new milestone on the PCSK9 road to tackle cardiovascular risk. Eur. Heart J. Cardiovasc. Pharmacother..

[100] Traber G.M., Yu A.M. (2024). The growing class of novel RNAi therapeutics. Mol. Pharmacol..

[101] Guo S., Zhang M., Huang Y. (2024). Three ‘E’ challenges for siRNA drug development. Trends Mol. Med..

[102] Padda I.S., Mahtani A.U., Patel P., Parmar M. (2026). Small Interfering RNA (siRNA) Therapy. StatPearls.

[103] Iwata-Endo K., Sahashi K., Kawai K., Fujioka Y., Okada Y., Watanabe E., Iwade N., Ishibashi M., Mohammad M., Aldoghachi A.F. (2025). Correcting tau isoform ratios with a long-acting antisense oligonucleotide alleviates 4R-tauopathy phenotypes. Mol. Ther. Nucleic Acids.

[104] Edwards A.L., Collins J.A., Junge C., Kordasiewicz H., Mignon L., Wu S., Li Y., Lin L., DuBois J., Hutchison R.M. (2023). Exploratory tau biomarker results from a multiple ascending-dose study of BIIB080 in Alzheimer disease: A randomized clinical trial. JAMA Neurol..

[105] Bennett C.F., Baker B.F., Pham N., Swayze E., Geary R.S. (2017). Pharmacology of antisense drugs. Annu. Rev. Pharmacol. Toxicol..

[106] Sud R., Geller E.T., Schellenberg G.D. (2014). Antisense-mediated exon skipping decreases tau protein expression: A potential therapy for tauopathies. Mol. Ther. Nucleic Acids.

[107] Shulman M., Wu S., Ziogas N., Edwards A., Collins J., Lin L., Tien I., Curiale G., Li Y., Mummery C. (2026). Exploratory analyses of clinical outcomes from the BIIB080 phase 1b study in mild Alzheimer’s disease. Nat. Aging.

[108] Harris G.A., Hirschfeld L.R. (2025). Antisense oligonucleotides provide optimism to the therapeutic landscape for tauopathies. Neural Regen. Res..

[109] Luo Y., Disney M.D. (2014). Bottom-up design of small molecules that stimulate exon 10 skipping in mutant MAPT pre-mRNA. ChemBioChem.

[110] Chen J.L., Zhang P., Abe M., Aikawa H., Zhang L., Frank A.J., Zembryski T., Hubbs C., Park H., Withka J. (2020). Design, optimization, and study of small molecules that target tau pre-mRNA and affect splicing. J. Am. Chem. Soc..

[111] Tang Z., Zhao J., Pearson Z.J., Boskovic Z.V., Wang J. (2021). RNA-targeting splicing modifiers: Drug development and screening assays. Molecules.

[112] Zhang P., Taghavi A., Abe M., Aikawa H., Akahori Y., Chen J.L., Tong Y., Baisden J.T., Cameron M.D., Childs-Disney J.L. (2025). Design of an orally bioavailable small molecule that modulates the microtubule-associated protein tau’s pre-mRNA splicing. ACS Chem. Biol..

[113] Ratni H., Ebeling M., Baird J., Bendels S., Bylund J., Chen K.S., Denk N., Feng Z., Green L., Guerard M. (2018). Discovery of risdiplam, a selective survival of motor neuron-2 (SMN2) gene splicing modifier for the treatment of spinal muscular atrophy (SMA). J. Med. Chem..

[114] Palacino J., Swalley S.E., Song C., Cheung A.K., Shu L., Zhang X., Van Hoosear M., Shin Y., Chin D.N., Gubser Keller C. (2015). SMN2 splice modulators enhance U1-pre-mRNA association and rescue SMA mice. Nat. Chem. Biol..

[115] Wang J., Schultz P.G., Johnson K.A. (2018). Mechanistic studies of a small-molecule modulator of SMN2 splicing. Proc. Natl. Acad. Sci. USA.

[116] Gao D., Morini E., Salani M., Krauson A.J., Chekuri A., Sharma N., Ragavendran A., Erdin S., Logan E.M., Li W. (2021). A deep learning approach to identify gene targets of a therapeutic for human splicing disorders. Nat. Commun..

[117] Angelbello A.J., Chen J.L., Disney M.D. (2020). Small molecule targeting of RNA structures in neurological disorders. Ann. N.Y. Acad. Sci..

[118] Salton M., Misteli T. (2016). Small molecule modulators of pre-mRNA splicing in cancer therapy. Trends Mol. Med..

[119] Morini E., Chekuri A., Logan E.M., Bolduc J.M., Kirchner E.G., Salani M., Krauson A.J., Narasimhan J., Gabbeta V., Grover S. (2023). Development of an oral treatment that rescues gait ataxia and retinal degeneration in a phenotypic mouse model of familial dysautonomia. Am. J. Hum. Genet..

[120] Rodriguez-Martin T., Garcia-Blanco M.A., Mansfield S.G., Grover A.C., Hutton M., Yu Q., Zhou J., Anderton B.H., Gallo J.M. (2005). Reprogramming of tau alternative splicing by spliceosome-mediated RNA trans-splicing: Implications for tauopathies. Proc. Natl. Acad. Sci. USA.

[121] Puttaraju M., Jamison S.F., Mansfield S.G., Garcia-Blanco M.A., Mitchell L.G. (1999). Spliceosome-mediated RNA trans-splicing as a tool for gene therapy. Nat. Biotechnol..

[122] Rodriguez-Martin T., Anthony K., Garcia-Blanco M.A., Mansfield S.G., Anderton B.H., Gallo J.M. (2009). Correction of tau mis-splicing caused by FTDP-17 *MAPT* mutations by spliceosome-mediated RNA trans-splicing. Hum. Mol. Genet..

[123] Avale M.E., Rodríguez-Martín T., Gallo J.M. (2013). Trans-splicing correction of tau isoform imbalance in a mouse model of tau mis-splicing. Hum. Mol. Genet..

[124] Muñiz J.A., Facal C.L., Urrutia L., Clerici-Delville R., Damianich A., Ferrario J.E., Falasco G., Avale M.E. (2022). SMaRT modulation of tau isoforms rescues cognitive and motor impairments in a preclinical model of tauopathy. Front. Bioeng. Biotechnol..

[125] Komor A.C., Kim Y.B., Packer M.S., Zuris J.A., Liu D.R. (2016). Programmable editing of a target base in genomic DNA without double-stranded DNA cleavage. Nature.

[126] Sousa A.A., Hemez C., Lei L., Traore S., Kulhankova K., Newby G.A., Doman J.L., Oye K., Pandey S., Karp P.H. (2025). Systematic optimization of prime editing for the efficient functional correction of CFTR F508del in human airway epithelial cells. Nat. Biomed. Eng..

[127] Roman A., Zimmer A., Gotthardt M., Steinmetz L.M., Kühn R., Dang T. (2025). Prime editing corrects the dilated cardiomyopathy causing RBM20-P633L-mutation in human cardiomyocytes. Mol. Ther. Nucleic Acids.

[128] Davis J.R., Banskota S., Levy J.M., Newby G.A., Wang X., Anzalone A.V., Nelson A.T., Chen P.J., Hennes A.D., An M. (2024). Efficient prime editing in mouse brain, liver and heart with dual AAVs. Nat. Biotechnol..

[129] Shirguppe S., Gapinske M., Swami D., Shenouda K., Miskalis A., Gosstola N., Del Bosque Siller D., Guerra I., Acharya P., Joulani D. (2026). In vivo CRISPR base editing for treatment of Huntington’s disease. Nat. Biomed. Eng..

[130] Xu X. (2026). Strategies of AAV capsid engineering for targeted delivery to brain, muscle, and retina. Front. Mol. Biosci..

[131] Donahue C.P., Muratore C., Wu J.Y., Kosik K.S. (2006). Stabilization of the MAPT exon 10-intron 10 regulatory stem-loop structure by structure-specific aminoglycoside binders. J. Biol. Chem..

[132] Varani L., Spillantini M.G., Goedert M., Varani G. (2000). Structural basis for recognition of the RNA major groove in the tau exon 10 splicing regulatory element by aminoglycoside antibiotics. Nucleic Acids Res..

[133] Yang L., Yin Y., Liu X., Guo B. (2026). Aptamer-Based Delivery of Genes and Drugs Across the Blood–Brain Barrier. Pharmaceuticals.

[134] Wang B., Pan X., Teng I.T., Li X., Kobeissy F., Wu Z.Y., Zhu J., Cai G., Yan H., Yan X. (2024). Functional selection of tau oligomerization-inhibiting aptamers. Angew. Chem. Int. Ed..

[135] Kim J.H., Kim E., Choi W.H., Lee J., Lee J.H., Lee H., Kim D.E., Suh Y.H., Lee M.J. (2016). Inhibitory RNA aptamers of tau oligomerization and their neuroprotective roles against proteotoxic stress. Mol. Pharm..

[136] Hanes J., Dobakova E., Majerova P. (2020). Brain drug delivery: Overcoming the blood-brain barrier to treat tauopathies. Curr. Pharm. Des..

[137] Khvorova A., Watts J.K. (2017). The chemical evolution of oligonucleotide therapies of clinical utility. Nat. Biotechnol..

[138] Scotti M.M., Swanson M.S. (2016). Pre-mRNA splicing in disease and therapeutics. Nat. Rev. Genet..

[139] Hammond S.M., Aartsma-Rus A., Alves S., Borgos S.E., Buijsen R.A.M., Collin R.W.J., Covello G., Denti M.A., Desviat L.R., Echevarría L. (2021). Delivery of oligonucleotide-based therapeutics: Challenges and opportunities. EMBO Mol. Med..

[140] ClinicalTrials.gov A First-In-Human Study of LY3954068 in Participants with Early Symptomatic Alzheimer’s Disease. Identifier NCT06297590. NCT06297590.

[141] ClinicalTrials.gov A Study to Evaluate the Efficacy of NIO752 in Participants with Progressive Supranuclear Palsy. Identifier NCT07498426. NCT07498426.

[142] Ziogas N., Mummery C.J., Rovira M.B., Cummings J.L., Fox N.C., Hansson O., Iwata A., Salloway S., Schneider A., Unwin A. (2023). Design of a phase 2, randomized, double-blind, placebo-controlled, parallel-group study to assess the efficacy, safety, and tolerability of BIIB080 in subjects with mild cognitive impairment due to Alzheimer’s disease or mild Alzheimer’s disease dementia. Alzheimer’s Dement..

[143] Biogen Inc. (2026). Biogen Presents Phase 2 CELIA Data at AAIC Demonstrating Meaningful Clinical Outcomes and Robust Tau Reduction with Diranersen in Early Alzheimer’s Disease. Biogen Investor Relations.

[144] De Vivo D.C., Bertini E., Swoboda K.J., Hwu W.L., Crawford T.O., Finkel R.S., Kirschner J., Kuntz N.L., Parsons J.A., Ryan M.M. (2019). Nusinersen initiated in infants during the presymptomatic stage of spinal muscular atrophy: Interim efficacy and safety results from the phase 2 NURTURE study. Neuromuscul. Disord..

[145] Iannaccone S.T., Paul D., Castro D., Weprin B., Swift D. (2021). Delivery of nusinersen through an Ommaya reservoir in spinal muscular atrophy. J. Clin. Neuromuscul. Dis..

[146] Schreiner T.G., Menéndez-González M., Schreiner O.D., Ciobanu R.C. (2025). Intrathecal therapies for neurodegenerative diseases: A review of current approaches and the urgent need for advanced delivery systems. Biomedicines.

[147] Strauss K.A., Carson V.J., Brigatti K.W., Young M., Robinson D.L., Hendrickson C., Fox M.D., Reed R.M., Puffenberger E.G., Mackenzie W. (2018). Preliminary safety and tolerability of a novel subcutaneous intrathecal catheter system for repeated outpatient dosing of nusinersen to children and adults with spinal muscular atrophy. J. Pediatr. Orthop..

[148] Shah P., Lalan M., Barve K. (2022). Intranasal delivery: An attractive route for the administration of nucleic acid based therapeutics for CNS disorders. Front. Pharmacol..

[149] Lipsman N., Meng Y., Bethune A.J., Huang Y., Lam B., Masellis M., Herrmann N., Heyn C., Aubert I., Boutet A. (2018). Blood–brain barrier opening in Alzheimer’s disease using MR-guided focused ultrasound. Nat. Commun..

[150] Janowicz P.W., Leinenga G., Götz J., Nisbet R.M. (2019). Ultrasound-mediated blood–brain barrier opening enhances delivery of therapeutically relevant formats of a tau-specific antibody. Sci. Rep..

[151] Silva M.C., Ferguson F.M., Cai Q., Donovan K.A., Nandi G., Patnaik D., Zhang T., Huang H.T., Lucente D.E., Dickerson B.C. (2019). Targeted degradation of aberrant tau in frontotemporal dementia patient-derived neuronal cell models. eLife.

[152] VandeVrede L., Boxer A.L., Polydoro M. (2020). Targeting tau: Clinical trials and novel therapeutic approaches. Neurosci. Lett..

[153] Thijssen E.H., La Joie R., Strom A., Fonseca C., Iaccarino L., Wolf A., Spina S., Allen I.E., Cobigo Y., Heuer H. (2021). Plasma phosphorylated tau 217 and phosphorylated tau 181 as biomarkers in Alzheimer’s disease and frontotemporal lobar degeneration: A retrospective diagnostic performance study. Lancet Neurol..

